# Hierarchical Ti_3_C_2_T_*x*_@ZnO Hollow Spheres with Excellent Microwave Absorption Inspired by the Visual Phenomenon of Eyeless Urchins

**DOI:** 10.1007/s40820-022-00817-5

**Published:** 2022-03-21

**Authors:** Yan-Qin Wang, Hai-Bo Zhao, Jin-Bo Cheng, Bo-Wen Liu, Qiang Fu, Yu-Zhong Wang

**Affiliations:** 1 Collaborative Innovation Center for Eco-Friendly and Fire-Safety Polymeric Materials (MoE), State Key Laboratory of Polymer Materials Engineering, National Engineering Laboratory for Eco-Friendly Polymer Materials (Sichuan), College of Chemistry, Sichuan University, Chengdu, 610064 People’s Republic of China; 2 College of Polymer Science and Engineering, State Key Laboratory of Polymer Materials Engineering, Sichuan University, Chengdu, 610065 People’s Republic of China

**Keywords:** Bioinspired, Hierarchical heterostructures, Ti_3_C_2_T_*x*_ MXene, ZnO nanoarrays, Microwave absorption

## Abstract

Ingenious microstructure design and rational composition selection are effective approaches to realize high-performance microwave absorbers, and the advancement of biomimetic manufacturing provides a new strategy. In nature, urchins are the animals without eyes but can “see”, because their special structure composed of regular spines and spherical photosensitive bodies “amplifies” the light-receiving ability. Herein, inspired by the above phenomenon, the biomimetic urchin-like Ti_3_C_2_T_*x*_@ZnO hollow microspheres are rationally designed and fabricated, in which ZnO nanoarrays (length: ~ 2.3 μm, diameter: ~ 100 nm) as the urchin spines are evenly grafted onto the surface of the Ti_3_C_2_T_*x*_ hollow spheres (diameter: ~ 4.2 μm) as the urchin spherical photosensitive bodies. The construction of gradient impedance and hierarchical heterostructures enhance the attenuation of incident electromagnetic waves. And the EMW loss behavior is further revealed by limited integral simulation calculations, which fully highlights the advantages of the urchin-like architecture. As a result, the Ti_3_C_2_T_*x*_@ZnO hollow spheres deliver a strong reflection loss of − 57.4 dB and broad effective absorption bandwidth of 6.56 GHz, superior to similar absorbents. This work provides a new biomimetic strategy for the design and manufacturing of advanced microwave absorbers.

**Supplementary Information:**

The online version contains supplementary material available at 10.1007/s40820-022-00817-5.

## Highlights

- Inspired by the photoreception mechanisms of urchins, the urchin-like Ti_3_C_2_T_*x*_@ZnO hollow spheres are rationally designed as high-performance microwave absorbents by constructing ZnO nanoarrays onto the surface of Ti_3_C_2_T_*x*_ hollow spheres.
- Both experimental and theoretical simulation results demonstrate that the microstructure of urchin-like possesses giant advantages in electromagnetic wave absorption performance, which is superior to most absorbers with similar components.

**Supplementary Information:**

The online version contains supplementary material available at 10.1007/s40820-022-00817-5.

## Graphical abstract

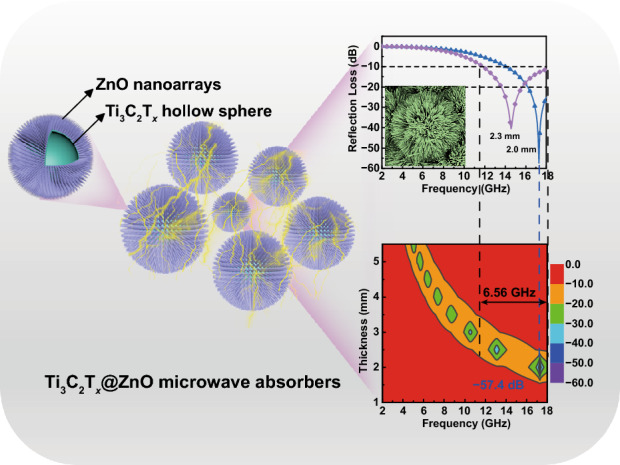

## Introduction

Recently, increasingly unwanted electromagnetic pollution has aggravated the demand for advanced microwave absorption (MA) materials [1–3]. Despite the fact that traditional microwave absorbers (e.g., carbon materials, ceramics, ferrites) have achieved satisfactory electromagnetic wave (EMW) absorption properties to some extent, their wide applications are still limited by the disadvantages of poor impedance matching, high density, narrow effective absorption bandwidth (EAB), inferior stability, etc. [4, 5]. In general, the design and fabrication of high-efficiency microwave absorbers mainly focus on the following two aspects [6, 7]. (i) The selection of material components. Dielectric loss and magnetic loss are well-known mechanisms for microwave absorbers design [8]. Up to now, various composite materials with enhanced MA performances have been obtained, including carbon-based composites [9–11], conductive polymer-based composites [12–14], ceramic-based composites [15, 16], as well as ferromagnetic composites [4, 17–19]. (ii) The construction of specific architecture. Various microstructures such as core–shell [20–22], yolk–shell [17, 23], foam and/or aerogel [5, 24, 25], and hollow structures [26–28] have been developed to obtain superior MA materials. Usually, these microstructures are considered to reduce the density and improve the impedance matching of microwave absorbers [29, 30].

Ti_3_C_2_T_*x*_ MXene, as a novel two-dimensional (2D) layered material, shows application potential in the MA field due to its special layered structure, high specific surface area, high electrical conductivity, and abundant terminal functional groups [31–33]. However, the exceeding high electrical conductivity of Ti_3_C_2_T_*x*_ MXene results in strong conduction loss, which tends to cause a mass of surface reflection of incident EMW [34–36]. Consequently, some effective strategies have been adopted to optimize the impedance matching to improve MA efficiency, such as surface modification, integration with other components, and rational design of microstructures. In addition, ZnO as a polarized semiconductor with excellent properties of dielectric, wide bandgap, long-term stability, and non-toxicity has attracted extensive attention in the field of MA [37]. Particularly for the oriented ZnO, the oriented polarization and planner defects greatly promote polarization loss of EMW and improve MA performance [38]. Accordingly, combining Ti_3_C_2_T_*x*_ MXene with oriented ZnO may be a great strategy to achieve high-performance EMW absorption through the reasonable microstructure design.

Advances in bio-inspiration and micro/nano manufacturing have made it possible to synthesize high-efficiency microwave absorbers through biomimetic microstructure design. In nature, urchins are reported to have no eyes but still possess the ability to “see”, the key to which may arise from their special structure consisting of regular spines and photoreceptors around the spherical bodies [39]. First, the spines of urchins are used as screening devices by screening off-axis light, thus giving themselves resolving vision. And higher spines density leads to more acuminous spatial vision [40]. Then, the residual light can be further transmitted and processed by the photoreceptor cells located around the spherical bodies of urchins [41]. As a result, the light receiving and detecting capabilities of urchins can be greatly “amplified”. This special photoreception enhancement mechanism of urchins offers inspirations for the design of high-efficiency microwave absorbers by taking advantages of urchin-like microstructures.

Inspired by the above photoreception enhancement behavior of urchins, we aim to design advanced EMW absorbing materials with enhanced MA performance. Herein, we have rationally designed and prepared urchin-like Ti_3_C_2_T_*x*_@ZnO hollow microspheres by mimicking the structure of urchins, in which ZnO nanoarrays (like urchin spines) are evenly grown on the surface of Ti_3_C_2_T_*x*_ MXene microspheres (like urchin spherical photosensitive bodies). In this design, regular ZnO nanoarrays with plenty of oxygen vacancies, defects, and abundance of void spaces can enhance the polarization, multiple reflection and scattering of incident EMW, thus playing the role of “shielding” EMW similar to the screening of incident light by urchin spines. And the Ti_3_C_2_T_*x*_ MXene hollow spheres, as urchin spherical bodies with photoreceptors, can further “conduct and process” the incident EMW that is not shielded by ZnO nanospines. Notes that, the Ti_3_C_2_T_*x*_ hollow spheres with high electrical conductivity can enhance the conduction loss of incident EMW, and the terminal functional groups induce local dipoles polarization to reinforce the attenuation of EMW. Further, the hollow structure of Ti_3_C_2_T_*x*_ MXene not only facilitates the multiple reflection and scattering of incident EMW but also improves the impedance matching. Relying on the above advantages and the construction of gradient impedance and hierarchical heterostructures between ZnO nanospines and Ti_3_C_2_T_*x*_ hollow spheres, the urchin-like hollow microspheres achieve a high-efficiency MA performance with a minimum reflection loss (RL_min_) of − 57.4 dB and EAB of 6.56 GHz, which is much superior to other absorbers with similar components. This work provides a significant inspiration for the biomimetic microstructure design of microwave absorbers.

## Experimental Section

### Fabrication of Ti_3_C_2_T_*x*_ Hollow Spheres

Ti_3_C_2_T_*x*_ MXene dispersion was prepared by selectively etching Al component of Ti_3_AlC_2_ MAX according to the previous report [42]. Typically, 1 g LiF powder was dissolved in 20 mL 9 M HCl, and then 1 g Ti_3_AlC_2_ powder was slowly added into the above solution with vigorous stirring. After stirring continuously for 24 h at 50 °C, the mixed solution was centrifuged and washed with deionized water several times until the pH reached neutral. Finally, the Ti_3_C_2_T_*x*_ MXene nanosheet dispersion was obtained by ultrasonication and centrifugation. Whereafter, the PMMA spheres ethanol dispersion was directly poured into the Ti_3_C_2_T_*x*_ MXene dispersion under stirring. The resulting solution was sonicated and stirred for 3 h. And the products marking as PMMA@Ti_3_C_2_T_*x*_ were collected by centrifugation at 3500 rpm and drying under vacuum at 80 °C for 2 h. The Ti_3_C_2_T_*x*_ hollow spheres were constructed via a sacrificial template method. Briefly, PMMA@Ti_3_C_2_T_*x*_ spheres were placed in a tubular furnace at 450, 550, and 650 °C for 90 min under N_2_ flow to obtain Ti_3_C_2_T_*x*_-450, Ti_3_C_2_T_*x*_-550, and Ti_3_C_2_T_*x*_-650, respectively.

### Fabrication of Urchin-Like Ti_3_C_2_T_*x*_@ZnO Hollow Spheres

ZnO nanoarrays were grown in situ on PMMA@Ti_3_C_2_T_*x*_ spheres via a facile hydrothermal reaction. In detail, 20 mg PMMA@Ti_3_C_2_T_*x*_ spheres were dispersed into 40 mL aqueous solution containing 2.8 mmol Zn(NO_3_)_2_·6H_2_O, and then 2.8 mmol hexamethylenetetramine was added into the above dispersion after stirring for 6 h. Subsequently, 1 mL ammonia was added to the above dispersion and stirred evenly. Then, the obtained mixture was transferred into a 100 mL Teflon-lined stainless-steel autoclave and heated at 105 °C for 16 h. The resulting precipitate was washed several times with deionized water and ethanol, dried at 80 °C, and named as PMMA@Ti_3_C_2_T_*x*_@ZnO. The preparation procedures of the Ti_3_C_2_T_*x*_@ZnO hollow spheres were completely consistent with Ti_3_C_2_T_*x*_ hollow spheres. The hollow spheres were respectively labeled as Ti_3_C_2_T_*x*_@ZnO-450, Ti_3_C_2_T_*x*_@ZnO-550, and Ti_3_C_2_T_*x*_@ZnO-650 after removing PMMA spheres by thermal treatment.

### Characterization

The phase composition and surface chemical valence state of the samples were recorded by powder X-ray diffraction (XRD, LabX XRD-6100, Shimadzu, Japan) and X-ray photoelectron spectroscopy (XPS, XSAM 800 spectrometer, Kratos Co., UK). Scanning electron microscope (SEM, Nova 600i) and transmission electron microscope (TEM, JEM-200CM, 20 kV) were employed to characterize the morphology and microstructure of the products. Fourier transform infrared (FTIR) spectra were performed by the Nicolet 6700 infrared spectrometer (Thermo electron corporation, USA). Thermogravimetry analysis was recorded by a Netzsch thermal analyzer (TGA 5500) in N_2_ atmosphere with a heating rate of 10 °C min^−1^. Electromagnetic parameters were measured by an Agilent N5230A vector network analyzer in the frequency range of 2−18 GHz. The measured samples were mixed with paraffin according to a certain mass fraction (5 wt% for Ti_3_C_2_T_*x*_ hollow spheres and 40 wt% for Ti_3_C_2_T_*x*_@ZnO hollow spheres) and pressed into a coaxial ring with an outer diameter of 7.00 mm and an inner diameter of 3.04 mm. The electric field distribution and electric energy loss distribution of the microwave absorbers are simulated and calculated by the limited integral method using the COMSOL software. The electromagnetic field in the simulation domain was obtained by solving Maxwell equations in the frequency domains, and the exact sizes of the samples were used in the numerical model construction.

## Results and Discussion

### Fabrication of Urchin-Like Ti_3_C_2_T_*x*_@ZnO Hollow Spheres

**Scheme 1:**
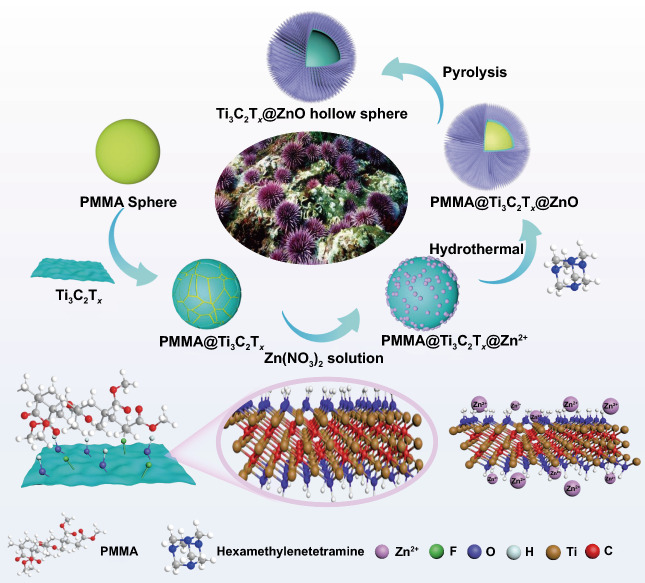
Schematic illustration of the synthesis process of urchin-like Ti_3_C_2_T_*x*_@ZnO hollow spheres

Scheme 1 illustrates the synthetic process of urchin-like Ti_3_C_2_T_*x*_@ZnO hollow spheres, which mainly adopts facile in situ self-assembly and template sacrifice strategies. 2D flexible Ti_3_C_2_T_*x*_ nanosheets are manufactured by selectively etching the Al layers in Ti_3_AlC_2_ MAX by the fluoride salt etching method (Fig. S1a), and the corresponding structural evolution from Ti_3_AlC_2_ MAX to Ti_3_C_2_T_*x*_ MXene nanosheets is displayed in Fig. S1b–e. The Ti_3_C_2_T_*x*_ nanosheets could be tightly attached to the surface of polymethyl methacrylate (PMMA) microspheres via hydrogen bonds and Van der Waals forces to form PMMA@Ti_3_C_2_T_*x*_ spheres [43]. Simultaneously, the terminal functional groups (−F, −OH, =O) of Ti_3_C_2_T_*x*_ MXene provide abundant deposition sites for Zn^2+^, which further facilitate the growth of ZnO nanoarrays to obtain the urchin-like PMMA@Ti_3_C_2_T_*x*_@ZnO microspheres. Then the composite spheres are further pyrolyzed to remove the PMMA spheres at varied temperatures under N_2_ atmosphere for 90 min to produce the ultimate urchin-like Ti_3_C_2_T_*x*_@ZnO hollow microspheres. Ti_3_C_2_T_*x*_ hollow microspheres as contrast are also prepared via a similar pyrolysis procedure.

**Fig. 1:**
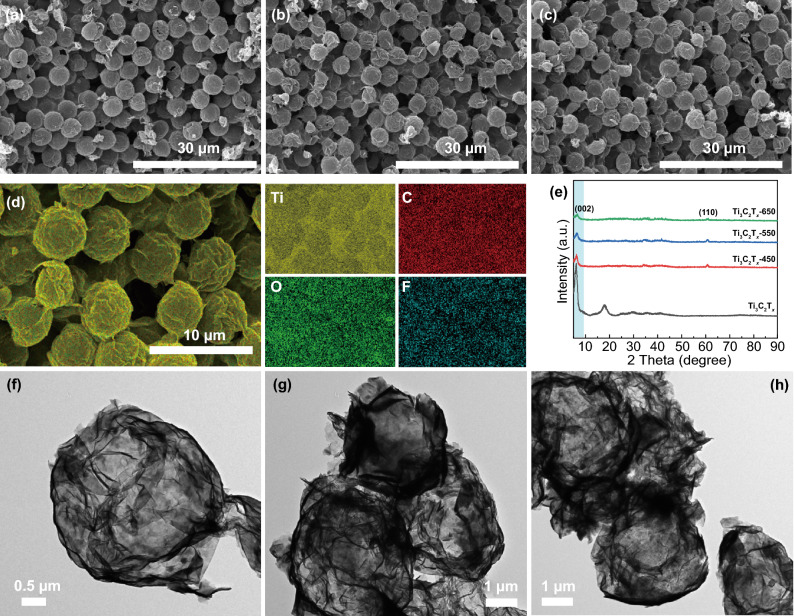
SEM images for **a** Ti_3_C_2_T_*x*_-450, **b** Ti_3_C_2_T_*x*_-550, and **c** Ti_3_C_2_T_*x*_-650; **d** the corresponding elemental mapping of Ti_3_C_2_T_*x*_-650; **e** XRD Patterns of Ti_3_C_2_T_*x*_ and Ti_3_C_2_T_*x*_ hollow spheres; TEM images for **f** Ti_3_C_2_T_*x*_-450, **g** Ti_3_C_2_T_*x*_-550, and **h** Ti_3_C_2_T_*x*_-650

The microstructure evolution and phase compositions for PMMA microspheres, PMMA@Ti_3_C_2_T_x_ composite spheres, and Ti_3_C_2_T_x_ hollow spheres are clearly illustrated in Figs. S2–S6 and 1. The surface of PMMA microspheres becomes rougher after being evenly coated with Ti_3_C_2_T_*x*_ nanosheets (Fig. S2). The strong hydrogen-bond interaction between PMMA and Ti_3_C_2_T_*x*_ can be confirmed by the fact that the FTIR characteristic peaks of PMMA@Ti_3_C_2_T_*x*_ generate red-shifts compared to those of PMMA (Fig. S3) [44]. Further, PMMA will be completely decomposed above 450 °C as shown in Fig. S4, while the structure and properties of Ti_3_C_2_T_*x*_ are also influenced by the high-temperature treatment. Therefore, PMMA@Ti_3_C_2_T_*x*_ composite microspheres are pyrolyzed at 450, 550, and 650 °C to achieve the Ti_3_C_2_T_*x*_ hollow spheres, named Ti_3_C_2_T_*x*_-450, Ti_3_C_2_T_*x*_-550, and Ti_3_C_2_T_*x*_-650, respectively. The resultant Ti_3_C_2_T_*x*_ hollow microspheres all maintain mutually independent spherical structure without collapse from the SEM images (Fig. 1a–d), indicating that 2D flexible Ti_3_C_2_T_*x*_ nanosheets possess a certain strength to form a self-supporting architecture after removing the PMMA spheres templates. And the interior hollow structure can be powerfully certified by TEM images (Fig. 1f–h), where the thin Ti_3_C_2_T_*x*_ nanosheets are assembled into uniform hollow spheres with a diameter of ~ 4.2 μm. Figures 1e and S5 indicate that all Ti_3_C_2_T_*x*_ hollow spheres exhibit the (002) characteristic peaks in the XRD patterns, however, the corresponding 2 Theta values gradually shift from 5.9° for the Ti_3_C_2_T_*x*_ nanoflakes to higher angles of 6.2°, 6.4°, and 6.5° for the Ti_3_C_2_T_*x*_-450, Ti_3_C_2_T_*x*_-550, and Ti_3_C_2_T_*x*_-650, respectively. In other words, the interlayer spacing decreases with the increase of pyrolysis temperature, which is attributed to the removal of intercalated water and surface termination groups of Ti_3_C_2_T_*x*_ [45]. X-ray photoelectron spectroscopy (XPS) spectra further reveal the evolution of the surface composition and chemical state from PMMA@Ti_3_C_2_T_*x*_ composite spheres to Ti_3_C_2_T_*x*_ hollow spheres. Figure S6a shows the absence of O−C=O in Ti_3_C_2_T_*x*_ hollow spheres compared with PMMA@Ti_3_C_2_T_*x*_, further indicating the complete pyrolysis of PMMA spheres [8, 46]. High-resolution XPS spectra (Fig. S6b, c) of O 1s and Ti 2p demonstrate that the contents of C–Ti–(OH)_*x*_ and O–C–(OH)_*x*_ decrease with increasing the pyrolysis temperature, accompanied by the increase of TiO_2_ content, which is caused by the departure of hydroxyl-containing functional groups [46–48]. The above structure evolution may have an influence on the dielectric properties of Ti_3_C_2_T_*x*_ hollow spheres, leading to different MA performance.

**Fig. 2:**
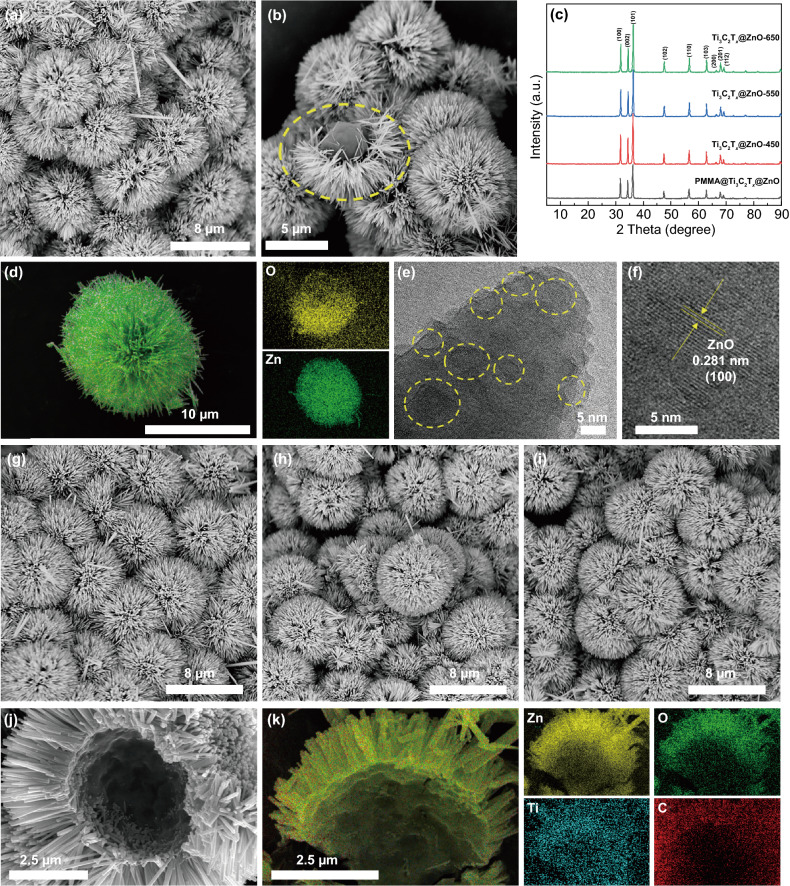
**a**, **b** SEM images for PMMA@Ti_3_C_2_T_*x*_@ZnO and **d** the corresponding elemental Mapping; **e**, **f** high-resolution TEM images for Ti_3_C_2_T_*x*_@ZnO-650; SEM images for **g** Ti_3_C_2_T_*x*_@ZnO-450, **h** Ti_3_C_2_T_*x*_@ZnO-550, and **i**, **j** Ti_3_C_2_T_*x*_@ZnO-650; **k** the elemental Mapping of Ti_3_C_2_T_*x*_@ZnO-650; **c** XRD patterns of PMMA@Ti_3_C_2_T_*x*_@ZnO and Ti_3_C_2_T_*x*_@ZnO hollow spheres

As the design, the urchin-like Ti_3_C_2_T_*x*_@ZnO composite microspheres are further fabricated by growing ZnO nanoarrays on Ti_3_C_2_T_*x*_ microspheres. However, ZnO nanoarrays cannot be directly grown on Ti_3_C_2_T_*x*_ hollow spheres due to the departure of functional groups on the surface of Ti_3_C_2_T_*x*_ MXene during the pyrolysis and collapse of the hollow structure caused by hydrothermal reaction. Consequently, ZnO nanoarrays as the urchin spines are first grown in situ on PMMA@Ti_3_C_2_T_*x*_ microspheres to obtain urchin-like PMMA@Ti_3_C_2_T_*x*_@ZnO (Fig. 2a, b). And the PMMA@Ti_3_C_2_T_*x*_ core can be clearly observed from the cracked PMMA@Ti_3_C_2_T_*x*_@ZnO spheres, marked with a yellow circle, as depicted in Fig. 2b. The energy dispersive spectrometer elemental Mapping confirms the uniform dispersion of Zn and O elements on PMMA@Ti_3_C_2_T_*x*_ spheres (Fig. 2d). It is worth noting that Ti_3_C_2_T_*x*_ MXene serving as Zn^2+^ deposition sites play a key role in the ZnO growth, while ZnO nanoarrays fail to be directly constructed on PMMA spheres due to the weak interface interaction (Fig. S7). After the growth of the ZnO nanoarrays, the PMMA cores can still be completely removed by the thermal cracking similar to those of PMMA@Ti_3_C_2_T_*x*_ microspheres (Figs. S4 and S8). Upon thermal treatment at 450, 550, and 650 °C, the urchin-like Ti_3_C_2_T_*x*_@ZnO hollow spheres are successfully manufactured. Figure 2g–i present the SEM images of Ti_3_C_2_T_*x*_@ZnO-450, Ti_3_C_2_T_*x*_@ZnO-550, and Ti_3_C_2_T_*x*_@ZnO-650, respectively. Compared with PMMA@Ti_3_C_2_T_*x*_@ZnO, Ti_3_C_2_T_*x*_@ZnO hollow microspheres have no apparent alterations in appearance, and still present the urchin-like architecture with an average diameter of ~ 8.8 μm. The structural evolution from PMMA@Ti_3_C_2_T_*x*_@ZnO to Ti_3_C_2_T_*x*_@ZnO spheres is more clearly revealed in Fig. 2j, k The SEM image of the cut Ti_3_C_2_T_*x*_@ZnO spheres verifies the typical hollow structure, where the ZnO nanospines with an average length of ~ 2.3 μm and a diameter of ~ 100 nm are evenly grown on the Ti_3_C_2_T_*x*_ cores with an average diameter of ~ 4.2 μm (Fig. 2j). Meanwhile, the elemental Mapping of Ti_3_C_2_T_*x*_@ZnO-650 displayed in Fig. 2k sufficiently attests that its smooth inner surface is composed of Ti_3_C_2_T_*x*_ nanoflake and the external spines are ZnO. This special hierarchical heterogeneous interface and hollow structure can facilitate superior MA performance. The cavities and defects emerge clearly on the ZnO nanospine (marked with yellow circles) as displayed in Fig. 2e, which can promote the polarization of incident EMW. And the lattice fringes as illustrated in Fig. 2f are well matched with the (100) plane of ZnO.

As-synthesized PMMA@Ti_3_C_2_T_*x*_@ZnO and Ti_3_C_2_T_*x*_@ZnO hollow spheres with distinct crystal structures are identified by XRD patterns (Fig. 2c). The diffraction peaks at 31.77°, 34.43°, 36.26°, 47.55°, 56.61°, 62.87°, 66.39°, 67.96°, and 69.11° are indexed to the (100), (002), (101), (102), (110), (103), (200), (201), and (112) crystal planes of the hexagonal wurtzite ZnO (PDF No. 89-0510), respectively. The absence of characteristic Ti_3_C_2_T_*x*_ peaks is attributed to the fact that the dense ZnO nanoarrays grown on the surface of Ti_3_C_2_T_*x*_ spheres shield the signal. XPS spectra are further applied to analyze the chemical state of Ti_3_C_2_T_*x*_@ZnO spheres. In the Zn 2p spectra (Fig. S9a), the peaks located at 1045.4 and 1022.2 eV correspond to Zn 2p_1/2_ and Zn 2p_3/2_, respectively [38, 49]. In Fig. S9b, with increasing thermal treatment temperature, the content of O atoms near oxygen vacancies (531.9 eV) increases evidently, which would promote the polarization behavior under microwave frequency. Further, according to the evolution of the surface chemical state of the Ti_3_C_2_T_*x*_ hollow spheres, it is reasonable to speculate that the Ti_3_C_2_T_*x*_ surface in Ti_3_C_2_T_*x*_@ZnO hollow spheres has undergone similar detachment of hydroxyl-containing groups. The evolution of ZnO nanoarrays and Ti_3_C_2_T_*x*_ spheres together affect the dielectric properties of Ti_3_C_2_T_*x*_@ZnO to achieve various EMW absorption properties.

### EMW Absorption Properties of Ti_3_C_2_T_*x*_@ZnO Hollow Spheres

The microwave absorption performance of the absorbers depends on the relative complex permittivity ( $\varepsilon _{{\text{r}}} \, = \,\varepsilon \prime \, - \,j\varepsilon ^{\prime\prime}$) and complex permeability ( $\mu _{{\text{r}}} \, = \,\mu ^{\prime}\, - \,{{j}}\mu ^{\prime\prime}$). Firstly, the electromagnetic parameters of Ti_3_C_2_T_*x*_ hollow spheres are measured in the frequency range of 2–18 GHz via the coaxial line method, which are presented in Fig. S10. The real parts of complex permittivity ( $\varepsilon \prime$) of Ti_3_C_2_T_*x*_ hollow spheres exhibit a declining trend with the increasing frequency, ascribing to the frequency dispersion behavior [50]. Taken as a whole, the higher pyrolysis temperature, the lower $\varepsilon \prime$ and $\varepsilon ^{\prime\prime}$ values of Ti_3_C_2_T_*x*_ hollow spheres. This decrease variation is due to the reduction in electrical conductivity according to the free electron theory [51]. The electrical conductivity of the Ti_3_C_2_T_*x*_ hollow spheres decreases with the increase of pyrolysis temperature on account of the loss of interlayer water and loosely adsorbed molecules on the surface, as well as the loss of Ti_3_C_2_T_*x*_ terminal functional groups and the formation of minor amounts of TiO_2_ crystal, resulting in the increase of pores volume [45]. Meanwhile, the dielectric loss tangent $\left( {\tan \delta _{e} \, = \,\varepsilon ^{\prime\prime}/\varepsilon ^{\prime}} \right)$ is applied to evaluate the dielectric loss capability, which is affected by both conduction loss and polarization loss [4, 52]. As illustrated in Fig. S10c, the Ti_3_C_2_T_*x*_-650 possesses the highest $\tan \delta _{{\text{e}}}$ value, which is the result of the competition between polarization loss and conduction loss. The decrease of $\tan \delta _{{\text{e}}}$ value indicates that the attenuated conduction loss plays a more important major role in dielectric loss when the thermal treatment temperature rises from 450 to 550 °C. Then the $\tan \delta _{{\text{e}}}$ values increase when the pyrolysis temperature is further up to 650 °C, confirming that the polarization loss becomes the major influencing factor. On the other hand, in view of the Ti_3_C_2_T_*x*_ hollow spheres as the dielectric loss-dominated material, the complex permeability value is close to 1 − *j*0, which will not be discussed.

**Fig. 3:**
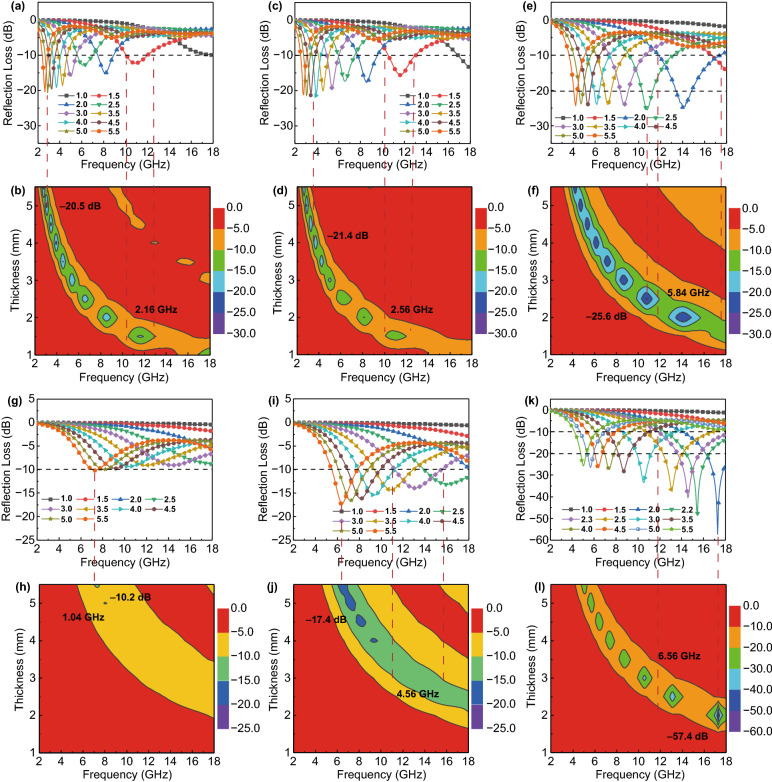
Frequency dependence of reflection loss for Ti_3_C_2_T_*x*_ hollow spheres and Ti_3_C_2_T_*x*_@ZnO hollow spheres: **a**, **b** Ti_3_C_2_T_*x*_-450, **c**, **d** Ti_3_C_2_T_*x*_-550, **e**, **f** Ti_3_C_2_T_*x*_-650, **g**, **h** Ti_3_C_2_T_*x*_@ZnO-450, **i**, **j** Ti_3_C_2_T_*x*_@ZnO-550, and **k**, **l** Ti_3_C_2_T_*x*_@ZnO-650

The MA performance is generally evaluated by the reflection loss (RL) and effective absorption bandwidth (EAB, the bandwidth of RL <  − 10 dB). The RL curves of Ti_3_C_2_T_*x*_ hollow microspheres are plotted in Fig. 3a–f, which can be calculated according to Eqs. S1 and S2. The RL_min_ value of Ti_3_C_2_T_*x*_-450 hollow spheres reaches to − 20.5 dB with the matching thickness of 5.0 mm, and the broadest EAB is 2.16 GHz at the thickness of 1.5 mm (Fig. 3a, b). The MA performance of Ti_3_C_2_T_*x*_-550 hollow spheres is not significantly improved compared with that of Ti_3_C_2_T_*x*_-450 (Fig. 3c, d). When the pyrolysis temperature is further increased to 650 °C, the RL_min_ value of Ti_3_C_2_T_*x*_-650 hollow spheres arrives to − 25.6 dB at 10.72 GHz with a thickness of 2.5 mm, and the broadest EAB can cover 5.84 GHz from 11.76 to 17.60 GHz with the thickness of 2.0 mm (Fig. 3e, f). The Ti_3_C_2_T_*x*_-650 hollow spheres exhibit relatively optimum MA properties stemming from the optimal dielectric loss capability and impedance matching, as confirmed in Figs. S10 and S11. The impedance matching is evaluated by the delta function that is described as Eq. S3. The calculated delta value should be as small as possible, which means that more incident EMW could propagate to the interior of absorbers without being substantially reflected on the surface. All the Ti_3_C_2_T_*x*_ hollow spheres thermally treated at various temperatures present relatively ideal impedance matching (Fig. S11). This benefits from the hollow structure that can induce more free space, thereby greatly improving the impedance matching. Although Ti_3_C_2_T_*x*_ MXene hollow spheres as urchin photoreceptors exhibit relatively good EMW absorption capacity to a certain extent, it remains the imperfection of the instability and high reflection of EMW caused by high electrical conductivity. As a consequence, according to our design, the coordination of “urchin spines” is necessary to further enhance the MA performance.

The frequency dependance of the complex permittivity of urchin-like Ti_3_C_2_T_*x*_@ZnO hollow spheres consisting of ZnO nanoarrays and Ti_3_C_2_T_*x*_ spheres are manifested in Fig. S12. Obviously, the $\varepsilon ^{\prime}$ and $\varepsilon ^{\prime\prime}$ values of Ti_3_C_2_T_x_@ZnO hollow microspheres gradually increase with the increase of thermal treatment temperature from 450 to 650 °C (Fig. S12a, b). More specifically, the average value of $\varepsilon ^{\prime}$ increases from 3.99 (Ti_3_C_2_T_x_@ZnO-450) to 4.70 (Ti_3_C_2_T_x_@ZnO-550), to 6.48 (Ti_3_C_2_T_x_@ZnO-650), while that of $\varepsilon ^{\prime\prime}$ value increases from 1.16 (Ti_3_C_2_T_x_@ZnO-450) to 1.79 (Ti_3_C_2_T_x_@ZnO-550), to 3.23 (Ti_3_C_2_T_x_@ZnO-650). The increased complex permittivity can usually be ascribed to two aspects, one is the increase in electrical conductivity, and the other is the enhancement in polarization. However, as discussed above, the electrical conductivity of Ti_3_C_2_T_x_ hollow spheres decreases with increasing pyrolysis temperature (Fig. S10). Therefore, the increased complex permittivity of Ti_3_C_2_T_x_@ZnO is mainly due to the enhanced polarization in defects and oxygen vacancies on ZnO nanospines. The Ti_3_C_2_T_x_@ZnO-650 exhibits the highest $\tan \delta _{{\text{e}}}$ value (Fig. S12c), demonstrating the strongest EMW dissipated capability. It’s worth noting that the complex permittivity curves of Ti_3_C_2_T_x_@ZnO hollow spheres have no such obvious resonant peaks compared with those of Ti_3_C_2_T_x_ hollow spheres. This resonant behavior in high frequency region is generally considered to be caused by high electrical conductivity and significant skin effect [53, 54].

Figure 3g–l depict the RL values of urchin-like Ti_3_C_2_T_*x*_@ZnO hollow microspheres with various thickness at the frequency range of 2–18 GHz. The RL values of Ti_3_C_2_T_*x*_@ZnO-450 are always ≥  − 10.2 dB, suggesting the negligible MA performance because of weak dielectric loss capability (Fig. 3g, h). When the pyrolysis temperature is increased to 550 °C, the MA performance of the composite microspheres is obviously enhanced. Specifically, the RL_min_ value reaches − 17.4 dB at 6.4 GHz with the matching thickness of 5.5 mm. And the broadest EAB arrives to 4.56 GHz from 15.68 to 11.12 GHz with the thickness of 3.0 mm (Fig. 3i, j). Nonetheless, the thick matching thickness makes it unable to satisfy the critical requirements of high-performance MA materials. As shown in Fig. 3k, l, it’s notable that when the matching thickness is only 2.0 mm, the RL_min_ value of Ti_3_C_2_T_*x*_@ZnO-650 hollow spheres reaches as strong as − 57.4 dB at 17.28 GHz (2.24 times that of Ti_3_C_2_T_*x*_ hollow spheres), accompanied by a broad EAB of 4.16 GHz (13.84–18 GHz). Moreover, the RL_min_ value can arrive to − 40.5 dB at 14.56 GHz with a thickness of 2.3 mm, while the EAB is as high as 6.56 GHz from 11.44 to 18 GHz, covering 41% of the entire measured frequency. By tunning the coating thickness from 2.0 to 5.5 mm, the RL_min_ values of Ti_3_C_2_T_*x*_@ZnO-650 hollow spheres are all less than − 20 dB, indicating that 99% of EMW can be dissipated in the corresponding frequency. Ultimately, both strong absorption capability and broad EAB are achieved. The MA capability and EAB can be effectively adjusted by constructing urchin-like core–shell heterojunctions and gradient impedance between Ti_3_C_2_T_*x*_ MXene and ZnO nanoarrays, as well as tailoring the thermal treatment temperature. Subsequently, the EMW absorption properties of the pure ZnO are investigated, as illustrated in Fig. S13. The RL_min_ is − 14.4 dB at 17.84 GHz and EAB is 1.84 GHZ from 16.16 to 18 GHz with a matching thickness of 1.5 mm. Overall, the MA performance of ZnO is inferior to those of Ti_3_C_2_T_*x*_ hollow spheres and Ti_3_C_2_T_*x*_@ZnO hollow spheres due to the weak dielectric properties (Fig. S14). All these results provide abundant evidence that the unique urchin-like architecture presents giant advantages for EMW absorption.

To understand the MA behavior of Ti_3_C_2_T_*x*_@ZnO hollow microspheres, the Cole–Cole semicircle curves are plotted to verify the polarization relaxation associated with complex permittivity according to the Deby theory (Eqs. S7–S10). As displayed in Fig. S15, the multiple semicircles in Cole–Cole curves of Ti_3_C_2_T_*x*_@ZnO-650 confirm the dielectric loss dominated by polarization loss. While those of Ti_3_C_2_T_*x*_@ZnO-450 and Ti_3_C_2_T_*x*_@ZnO-550 exhibit multiple semicircles with a linear tail, indicating the simultaneous presence of polarization relaxation and conduction loss. By increasing the pyrolysis temperature, the enhancement of polarization loss is greater than conduction loss. The impedance matching of urchin-like Ti_3_C_2_T_*x*_@ZnO-650 hollow microspheres is further investigated in Fig. S16. The hollow structure of Ti_3_C_2_T_*x*_ spheres and the void spaces between ZnO spines effectively improve the impedance matching of the composites. By contrast, the increase of thermal treatment temperature is in favor of further improving the impedance matching of Ti_3_C_2_T_*x*_@ZnO hollow spheres due to the enhancement of polarization loss. The attenuation constant α indicates the capability of microwave absorbers to convert the EMW to thermal and/or other forms of energy, which can be described as Eq. S6. Comparatively, Ti_3_C_2_T_*x*_@ZnO-650 hollow spheres show the highest α value (Fig. S17), which represents the strongest EMW attenuation ability. The superior EMW attenuation ability of Ti_3_C_2_T_*x*_@ZnO is mainly derived from the strong polarization loss caused by defect-rich ZnO spines, moderate conduction loss from Ti_3_C_2_T_*x*_ hollow spheres, and interface polarization loss. Both the optimal impedance matching and strong microwave attenuation capability make Ti_3_C_2_T_*x*_@ZnO-650 for the high-efficiency broadband MA performance.

**Fig. 4:**
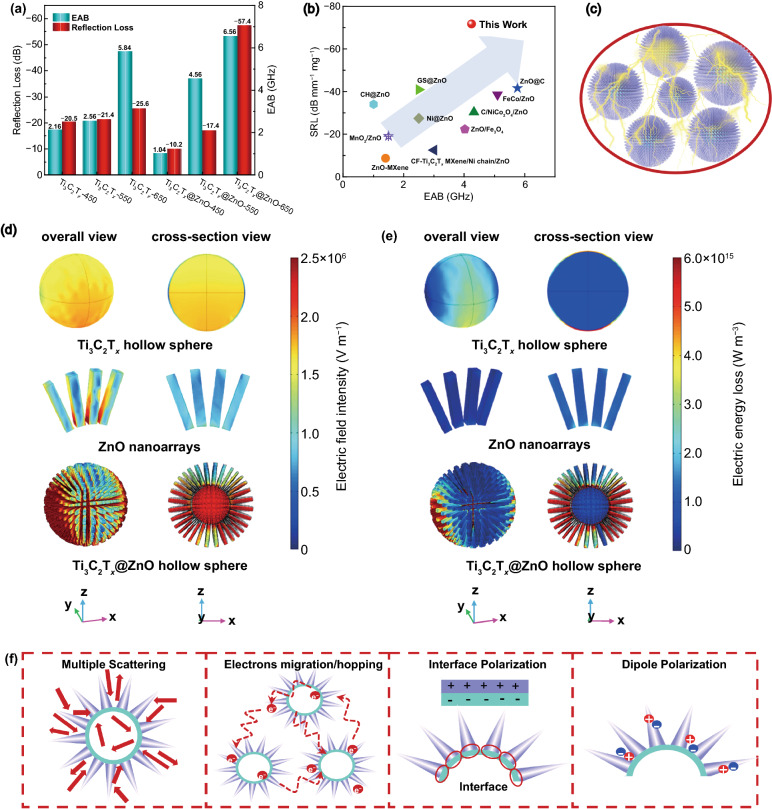
**a** Comparison of RL_min_ and EAB of Ti_3_C_2_T_*x*_ hollow spheres and urchin-like Ti_3_C_2_T_*x*_@ZnO hollow spheres; **b** SRL and EAB of ZnO-based composites; **d** Complex electric field intensity distribution and **e** electric energy loss distribution; **c**, **f** Schematic illustration of microwave absorption mechanisms for urchin-like Ti_3_C_2_T_*x*_@ZnO hollow microspheres

The RL_min_ values and the broadest EAB for Ti_3_C_2_T_*x*_ hollow spheres and urchin-like Ti_3_C_2_T_*x*_@ZnO hollow spheres obtained at various temperatures are summarized in Fig. 4a for a comparative analysis of the MA properties. On the whole, 650 °C is the most suitable pyrolysis temperature for the fabrication of the Ti_3_C_2_T_*x*_ and Ti_3_C_2_T_*x*_@ZnO hollow spheres. The optimum MA performance of Ti_3_C_2_T_*x*_-650 is evidenced by − 25.6 dB for RL_min_ and 5.84 GHz for the broadest EAB. And − 57.4 dB of RL_min_ and 6.56 GHz of the broadest EAB are achieved for Ti_3_C_2_T_*x*_@ZnO-650. Both the RL_min_ value and the EAB of the urchin-like Ti_3_C_2_T_*x*_@ZnO hollow microspheres are much smaller and broader in comparison with those of the Ti_3_C_2_T_*x*_ hollow microspheres, suggesting that the construction of biomimetic urchin structure possesses a vital influence on the EMW absorption capability. In general, the superior microwave absorbers should possess thin–matching thickness, light–weight, broad EAB, and strong MA intensity, simultaneously [55]. In consequence, Yu et al. proposed the specific reflection loss (SRL) to evaluate the MA performance of microwave absorbers more clearly, which can be calculated according to Eq. S11 [56]. Figure 4b compares the SRL values and the corresponding EAB of the ZnO-based composite microwave absorbers reported in previous literatures, and the corresponding specific values are listed in Table S1. By contrast, the Ti_3_C_2_T_*x*_@ZnO-650 presents a relatively high SRL value and broad EAB, indicating that the urchin-like Ti_3_C_2_T_*x*_@ZnO hollow spheres take on a bright application prospect as a kind of light-weight, ultrathin, and high-efficiency MA materials.

### EMW Absorption Mechanisms Analysis of Ti_3_C_2_T_*x*_@ZnO Hollow Spheres

In order to intuitively explore the influencing mechanisms of urchin-like architecture on MA, the electric field intensity distribution and electric energy loss distribution of Ti_3_C_2_T_*x*_ hollow spheres, ZnO nanoarrays, and Ti_3_C_2_T_*x*_@ZnO hollow spheres are simulated by the limited integral method (Fig. 4d, e). For the Ti_3_C_2_T_*x*_ hollow spheres, the electric field is concentrated inside the hollow structure, indicating that part of the microwave can propagate to the interior of Ti_3_C_2_T_*x*_ hollow spheres, and be repeatedly reflected and scattered in the cavity to excite the electrons on the Ti_3_C_2_T_*x*_ surface. At the same time, when EMW passes through MXene, it will interact with the high-density electrons of MXene to induce electric current, which leads to a certain power loss in EMW. As for the pure ZnO nanoarrays, the electric field intensity distribution is relatively weak on the whole, and the electric energy loss capacity is almost negligible. Interestingly, when ZnO nanoarrays and MXene spheres are assembled into unique urchin-like architecture, significant electric field concentration can be both found in the exterior nanospines and interior hollow spheres. And the corresponding electric energy loss capabilities are also largely improved (Fig. 4e). On one hand, the gradient impedance formed after the growth of the ZnO nanoarrays on the surface of Ti_3_C_2_T_*x*_ hollow spheres allows more EMW to propagate into the composite, and the repeated reflection and scattering occurred on the hierarchical interfaces extend transmission path of EMW to achieve more energy dissipation. On the other hand, it can be observed that the power loss of Ti_3_C_2_T_*x*_@ZnO is still intense in the external ZnO spines region, which is rather different from the pure ZnO nanoarrays. A key reason for this phenomenon should be the disparity of electron motion between them, while the excited electrons in Ti_3_C_2_T_*x*_ migrate along the axis or jump to the adjacent ZnO spines through interfaces, defects, etc., thereby significantly promoting the electric energy loss. The simulation calculation results provide compelling evidence that the unique urchin-like architecture has a vital positive effect on enhancing EMW consumption.

Based on the above experiment and theoretical simulation results, the involved MA mechanisms of the urchin-like Ti_3_C_2_T_*x*_@ZnO hollow microspheres are revealed as illustrated in Fig. 4c–f. When the EMW is incident into the Ti_3_C_2_T_*x*_@ZnO hollow microspheres absorbers (Fig. 4c), the attenuation and dissipation of EMW are mainly derived from the synergistic reinforcing effect between ZnO nanospines, Ti_3_C_2_T_*x*_ MXene hollow cores, and hierarchical urchin-like heterostructures. Specifically, the hollow structure of Ti_3_C_2_T_*x*_ cores, the abundance of void spaces among ZnO nanospines, and the construction of gradient impedance and hierarchical heterostructures can promote the multiple reflection and scattering of incident EMW, extend its propagation routes, and reinforce the dissipation of the EMW [57]. Meanwhile, the hollow structure and void spaces can also be used as the impedance matching mediator to balance the impedance of Ti_3_C_2_T_*x*_@ZnO and air [4, 58]. Secondly, under the alternating electromagnetic field, the excited electrons in Ti_3_C_2_T_*x*_ hollow spheres migrate along the axial direction or hop to the neighboring ZnO spines through the defects, interfaces, heterojunctions, and conductive network, finally converting the EMW into thermal energy [38, 59]. Thirdly, the urchin-like Ti_3_C_2_T_*x*_@ZnO hollow microspheres provide plenty of heterogeneous interfaces, which leads to the accumulation of free electrons at interfaces to produce interface polarization [54, 60]. The terminal functional groups on Ti_3_C_2_T_*x*_ MXene cores and the defects and oxygen vacancies on ZnO spines can induce local dipoles polarization [61]. In short, the high-efficiency MA performance of the biomimetic urchin-like Ti_3_C_2_T_*x*_@ZnO hollow microspheres benefits from the synergistic effects of the ZnO nanospines, Ti_3_C_2_T_*x*_ MXene hollow spheres, and specific urchin-like architecture on incident EMW.

## Conclusions

In summary, the biomimetic urchin-like Ti_3_C_2_T_*x*_@ZnO hollow microspheres are designed and constructed for the first time by the in situ self-assembly and template sacrificial methods according to the photoreception principles of urchins. The ZnO nanospines with an average length of ~ 2.3 μm and diameter of ~ 100 nm have been successfully grown on the Ti_3_C_2_T_*x*_ cores with an average diameter of ~ 4.2 μm. In the urchin-like hollow spheres, the ZnO nanospines primarily “shield” the incident EMW, while the Ti_3_C_2_T_*x*_ hollow spheres further “conduct and process” the EMW that cannot be dissipated by ZnO spines. The giant advantages of urchin-like architecture on the EMW consumption are innovatively revealed by the limited integral simulation, which greatly boosts the power loss capacity. Consequently, the urchin-like Ti_3_C_2_T_*x*_@ZnO hollow spheres exhibit excellent MA performance with the absorption of as strong as − 57.4 dB and EAB of as broad as 6.56 GHz. The experimental and theoretical simulation results demonstrate that the construction of oriented ZnO nanospines, highly conductive Ti_3_C_2_T_*x*_ cores, hollow structures, gradient impedance, as well as hierarchical urchin-like heterostructures are conducive to the manufacture of high-performance MA composites. Therefore, it’s believed that this work will provide a reference for the design and construction of high-efficiency microwave absorbers with biomimetic hierarchical microstructures in the future.

## Supplementary Information

Below is the link to the electronic supplementary material.

Supplementary file1 (PDF 2091 kb)

## References

[1] Song P, Liu B, Liang C, Ruan K, Qiu H (2021). Lightweight, flexible cellulose-derived carbon aerogel@reduced graphene oxide/PDMS composites with outstanding EMI shielding performances and excellent thermal conductivities. Nano-Micro Lett..

[2] Zeng Z, Jiang F, Yue Y, Han D, Lin L (2020). Flexible and ultrathin waterproof cellular membranes based on high-conjunction metal-wrapped polymer nanofibers for electromagnetic interference shielding. Adv. Mater..

[3] Zhu Y, Liu J, Guo T, Wang JJ, Tang X (2021). Multifunctional Ti_3_C_2_T_x_ MXene composite hydrogels with strain sensitivity toward absorption-dominated electromagnetic-interference shielding. ACS Nano.

[4] Wang S, Li D, Zhou Y, Jiang L (2020). Hierarchical Ti_3_C_2_T_x_ MXene/Ni chain/ZnO array hybrid nanostructures on cotton fabric for durable self-cleaning and enhanced microwave absorption. ACS Nano.

[5] Liang L, Li Q, Yan X, Feng Y, Wang Y (2021). Multifunctional magnetic Ti_3_C_2_T_x_ MXene/graphene aerogel with superior electromagnetic wave absorption performance. ACS Nano.

[6] Wang Y, Zhong W, Zhang S, Zhang X, Zhu C (2022). Pearl necklace-like CoMn-based nanostructures derived from metal-organic frames for enhanced electromagnetic wave absorption. Carbon.

[7] Pang H, Duan Y, Huang L, Song L, Liu J (2021). Research advances in composition, structure and mechanisms of microwave absorbing materials. Compos. B Eng..

[8] Wang J, Liu L, Jiao S, Ma K, Lv J (2020). Hierarchical carbon fiber@MXene@MoS_2_ core-sheath synergistic microstructure for tunable and efficient microwave absorption. Adv. Funct. Mater..

[9] Song Q, Ye F, Kong L, Shen Q, Han L (2020). Graphene and MXene nanomaterials: toward high-performance electromagnetic wave absorption in gigahertz band range. Adv. Funct. Mater..

[10] Lu Y, Zhang S, He M, Wei L, Chen Y (2021). 3D cross-linked graphene or/and MXene based nanomaterials for electromagnetic wave absorbing and shielding. Carbon.

[11] Cheng JB, Wang YQ, Zhang AN, Zhao HB, Wang YZ (2021). Growing MoO_3_-doped WO_3_ nanoflakes on rGO aerogel sheets towards superior microwave absorption. Carbon.

[12] Ding J, Wang L, Zhao Y, Xing L, Yu X (2019). Boosted interfacial polarization from multishell TiO_2_@Fe_3_O_4_@PPy heterojunction for enhanced microwave absorption. Small.

[13] He N, Yang X, Shi L, Yang X, Lu Y (2020). Chemical conversion of Cu_2_O/PPy core-shell nanowires (CSNWs): a surface/interface adjustment method for high-quality Cu/Fe/C and Cu/Fe_3_O_4_/C CSNWs with superior microwave absorption capabilities. Carbon.

[14] Xu C, Wu F, Duan L, Xiong Z, Xia Y (2020). Dual-interfacial polarization enhancement to design tunable microwave absorption nanofibers of SiC@C@PPy. ACS Appl. Electron. Mater..

[15] Lu MM, Cao WQ, Shi HL, Fang XY, Yang J (2014). Multi-wall carbon nanotubes decorated with ZnO nanocrystals: mild solution-process synthesis and highly efficient microwave absorption properties at elevated temperature. J. Mater. Chem. A.

[16] Yan L, Hong C, Sun B, Zhao G, Cheng Y (2017). In situ growth of core–sheath heterostructural SiC nanowire arrays on carbon fibers and enhanced electromagnetic wave absorption performance. ACS Appl. Mater. Interfaces.

[17] Liu Q, Cao Q, Bi H, Liang C, Yuan K (2016). CoNi@SiO_2_@TiO_2_ and CoNi@Air@TiO_2_ microspheres with strong wideband microwave absorption. Adv. Mater..

[18] Zhao B, Li Y, Zeng Q, Wang L, Ding J (2020). Galvanic replacement reaction involving core-shell magnetic chains and orientation-tunable microwave absorption properties. Small.

[19] Ma X, Duan Y, Huang L, Lei H, Yang X (2022). Quasiperiodic metamaterials with broadband absorption: tailoring electromagnetic wave by Penrose tiling. Compos. B Eng..

[20] Wen B, Yang H, Lin Y, Ma L, Qiu Y (2021). Synthesis of core–shell Co@S-doped carbon@mesoporous N-doped carbon nanosheets with a hierarchically porous structure for strong electromagnetic wave absorption. J. Mater. Chem. A.

[21] Cui E, Pan F, Xiang Z, Liu Z, Yu L (2020). Engineering dielectric loss of FeCo/polyvinylpyrrolidone core-shell nanochains@graphene oxide composites with excellent microwave absorbing properties. Adv. Eng. Mater..

[22] Yang PA, Huang Y, Li R, Huang X, Ruan H (2022). Optimization of Fe@Ag core–shell nanowires with improved impedance matching and microwave absorption properties. Chem. Eng. J..

[23] Li H, Bao S, Li Y, Huang Y, Chen J (2018). Optimizing the electromagnetic wave absorption performances of designed Co_3_Fe_7_@C yolk–shell structures. ACS Appl. Mater. Interfaces.

[24] Zhu M, Yan X, Xu H, Xu Y, Kong L (2021). Ultralight, compressible, and anisotropic MXene@wood nanocomposite aerogel with excellent electromagnetic wave shielding and absorbing properties at different directions. Carbon.

[25] Cheng Y, Zhao H, Lv H, Shi T, Ji G (2020). Lightweight and flexible cotton aerogel composites for electromagnetic absorption and shielding applications. Adv. Electron. Mater..

[26] Liu P, Gao S, Zhang G, Huang Y, You W (2021). Hollow engineering to Co@N-doped carbon nanocages via synergistic protecting-etching strategy for ultrahigh microwave absorption. Adv. Funct. Mater..

[27] Wen G, Zhao X, Liu Y, Zhang H, Wang C (2019). Facile synthesis of RGO/Co@Fe@Cu hollow nanospheres with efficient broadband electromagnetic wave absorption. Chem. Eng. J..

[28] Wang HY, Sun XB, Yang SH, Zhao PY, Zhang XJ (2021). 3D ultralight hollow NiCo compound@MXene composites for tunable and high-efficient microwave absorption. Nano-Micro Lett..

[29] Zeng Z, Mavrona E, Sacré D, Kummer N, Cao J (2021). Terahertz birefringent biomimetic aerogels based on cellulose nanofibers and conductive nanomaterials. ACS Nano.

[30] Liang C, Qiu H, Song P, Shi X, Kong J (2020). Ultra-light MXene aerogel/wood-derived porous carbon composites with wall-like “mortar/brick” structures for electromagnetic interference shielding. Sci. Bull..

[31] Zeng Z, Wang C, Siqueira G, Han D, Huch A (2020). Nanocellulose-MXene biomimetic aerogels with orientation-tunable electromagnetic interference shielding performance. Adv. Sci..

[32] Zhang Y, Ruan K, Gu J (2021). Flexible sandwich-structured electromagnetic interference shielding nanocomposite films with excellent thermal conductivities. Small.

[33] Shin H, Eom W, Lee KH, Jeong W, Kang DJ (2021). Highly electroconductive and mechanically strong Ti_3_C_2_T_x_ MXene fibers using a deformable MXene gel. ACS Nano.

[34] Kong L, Qi J, Li M, Chen X, Yuan X (2021). Electromagnetic wave absorption properties of Ti_3_C_2_T_x_ nanosheets modified with in-situ growth carbon nanotubes. Carbon.

[35] Liu LX, Chen W, Zhang HB, Wang QW, Guan F (2019). Flexible and multifunctional silk textiles with biomimetic leaf-like MXene/silver nanowire nanostructures for electromagnetic interference shielding, humidity monitoring, and self-derived hydrophobicity. Adv. Funct. Mater..

[36] Liang C, Gu Z, Zhang Y, Ma Z, Qiu H (2021). Structural design strategies of polymer matrix composites for electromagnetic interference shielding: a review. Nano-Micro Lett..

[37] Ma W, Yang R, Wang T (2020). ZnO Nanorod-based microflowers decorated with Fe_3_O_4_ nanoparticles for electromagnetic wave absorption. ACS Appl. Nano Mater..

[38] Wang L, Li X, Li Q, Yu X, Zhao Y (2019). Oriented polarization tuning broadband absorption from flexible hierarchical ZnO arrays vertically supported on carbon cloth. Small.

[39] Kirwan JD, Bok MJ, Smolka J, Foster JJ, Hernandez JC (2018). The sea urchin *Diadema africanum* uses low resolution vision to find shelter and deter enemies. J. Exp. Biol..

[40] Yerramilli D, Johnsen S (2010). Spatial vision in the purple sea urchin *Strongylocentrotus purpuratus* (Echinoidea). J. Exp. Biol..

[41] Ullrich-Luter EM, Dupont S, Arboleda E, Hausen H, Arnone MI (2011). Unique system of photoreceptors in sea urchin tube feet. PNAS.

[42] Wang Y, Liu R, Zhang J, Miao M, Feng X (2021). Vulcanization of Ti_3_C_2_T_*x*_ MXene/natural rubber composite films for enhanced electromagnetic interference shielding. Appl. Surf. Sci..

[43] Li X, Yin X, Song C, Han M, Xu H (2018). Self-assembly core-shell graphene-bridged hollow MXenes spheres 3D foam with ultrahigh specific EM absorption performance. Adv. Funct. Mater..

[44] Liang L, Han G, Li Y, Zhao B, Zhou B (2019). Promising Ti_3_C_2_T_*x*_ MXene/Ni chain hybrid with excellent electromagnetic wave absorption and shielding capacity. ACS Appl. Mater. Interfaces.

[45] Iqbal A, Shahzad F, Hantanasirisakul K, Kim M, Kwon J (2020). Anomalous absorption of electromagnetic waves by 2D transition metal carbonitride Ti_3_CNTx (MXene). Science.

[46] Yang M, Yuan Y, Li Y, Sun X, Wang S (2020). Anisotropic electromagnetic absorption of aligned Ti_3_C_2_T_x_ MXene/gelatin nanocomposite aerogels. ACS Appl. Mater. Interfaces.

[47] Kong M, Jia Z, Wang B, Dou J, Liu X (2020). Construction of metal-organic framework derived Co/ZnO/Ti_3_C_2_T_x_ composites for excellent microwave absorption. Sustain. Mater. Technol..

[48] Li S, Wang J, Zhu Z, Liu D, Li W (2021). CVD carbon-coated carbonized loofah sponge loaded with a directionally arrayed MXene aerogel for electromagnetic interference shielding. J. Mater. Chem. A.

[49] Li X, Wang L, You W, Li X, Yang L (2019). Enhanced microwave absorption performance from abundant polarization sites of ZnO nanocrystals embedded in CNTs via confined space synthesis. Nanoscale.

[50] Zhou X, Jia Z, Feng A, Wang K, Liu X (2020). Dependency of tunable electromagnetic wave absorption performance on morphology-controlled 3D porous carbon fabricated by biomass. Compos. Commun..

[51] Qiang R, Du Y, Zhao H, Wang Y, Tian C (2015). Metal organic framework-derived Fe/C nanocubes toward efficient microwave absorption. J. Mater. Chem. A.

[52] Quan B, Shi W, Ong SJH, Lu X, Wang PL (2019). Defect engineering in two common types of dielectric materials for electromagnetic absorption applications. Adv. Funct. Mater..

[53] Liu Q, Cao B, Feng C, Zhang W, Zhu S (2012). High permittivity and microwave absorption of porous graphitic carbons encapsulating Fe nanoparticles. Compos. Sci. Technol..

[54] Wang Y, Wang H, Ye J, Shi L, Feng X (2020). Magnetic CoFe alloy@C nanocomposites derived from ZnCo-MOF for electromagnetic wave absorption. Chem. Eng. J..

[55] Zhang Z, Cai Z, Zhang Y, Peng Y, Wang Z (2021). The recent progress of MXene-based microwave absorption materials. Carbon.

[56] Li Y, Liu X, Nie X, Yang W, Wang Y (2019). Multifunctional organic–inorganic hybrid aerogel for self-cleaning, heat-insulating, and highly efficient microwave absorbing material. Adv. Funct. Mater..

[57] Liu J, Zhang HB, Sun R, Liu Y, Liu Z (2017). Hydrophobic, flexible, and lightweight MXene foams for high-performance electromagnetic-interference shielding. Adv. Mater..

[58] Yan L, Zhang M, Zhao S, Sun T, Zhang B (2020). Wire-in-tube ZnO@carbon by molecular layer deposition: accurately tunable electromagnetic parameters and remarkable microwave absorption. Chem. Eng. J..

[59] Cheng JB, Zhao HB, Cao M, Li ME, Zhang AN (2020). Banana leaflike C-doped MoS_2_ aerogels toward excellent microwave absorption performance. ACS Appl. Mater. Interfaces.

[60] Zhao HB, Cheng JB, Wang YZ (2018). Biomass-derived Co@ crystalline carbon@ carbon aerogel composite with enhanced thermal stability and strong microwave absorption performance. J. Alloys Compd..

[61] Zhang XJ, Zhu JQ, Yin PG, Guo AP, Huang AP (2018). Tunable high-performance microwave absorption of Co_1__−__x_S hollow spheres constructed by nanosheets within ultralow filler loading. Adv. Funct. Mater..

