# Supplementary material for: Hierarchical Ti3C2Tx@ZnO Hollow Spheres with Excellent Microwave Absorption Inspired by the Visual Phenomenon of Eyeless Urchins
Source: Nanomicro Lett. 2022 Mar 21;14:76. doi: 10.1007/s40820-022-00817-5 (PMC8938554; doi:10.1007/s40820-022-00817-5)
Supplement: Supplementary file 1 — Supplementary file1 (PDF 2091 kb) [file 40820_2022_817_MOESM1_ESM.pdf]

Supporting Information for

Hierarchical $Ti_3C_2T_x$@ZnO Hollow Spheres with Excellent Microwave Absorption Inspired by the Visual Phenomenon of Eyeless Urchins

Yan-Qin Wang[1], Hai-Bo Zhao[1,]*, Jin-Bo Cheng[1], Bo-Wen Liu[1], Qiang Fu[2], Yu-Zhong Wang[1,]*

[1]Collaborative Innovation Center for Eco-Friendly and Fire-Safety Polymeric Materials (MoE), State Key Laboratory of Polymer Materials Engineering, National Engineering Laboratory for Eco-Friendly Polymer Materials (Sichuan), College of Chemistry, Sichuan University, Chengdu 610064, P. R. China

[2]College of Polymer Science & Engineering, State Key Laboratory of Polymer Materials Engineering, Sichuan University, Chengdu 610065, P. R. China

*Corresponding authors. E-mail: haibor7@163.com (Hai-Bo Zhao); yzwang@scu.edu.cn (Yu-Zhong Wang)

S1 Supplementary Text

S1.1 Microwave Absorption (MA) Measurements

The MA performance is generally evaluated by the reflection loss (RL) and effective absorption bandwidth (EAB, the bandwidth of RL<−10 dB). The RL can be calculated according transmission theory as follows [S1, S2]:

$$Z_{in} = Z_0\sqrt{\frac{\mu_r}{\varepsilon_r}}\tan h\left[j\left(\frac{2\pi f d}{c}\right)\sqrt{\mu_r\varepsilon_r}\right] \quad \text{(S1)}$$

$$RL = 20log_{10}\left|\frac{Z_{in}-Z_0}{Z_{in}+Z_0}\right| \quad \text{(S2)}$$

Where Z_{in} is the input impedance of the microwave absorbers, Z_0 is the impedance of free space, f is the frequency of microwave, d is the thickness of the absorbers, c is the velocity of light in free space, ε_r ($\varepsilon_r = \varepsilon' - j\varepsilon''$) and μ_r ($\mu_r = \mu' - j\mu''$) refer to the complex permittivity and complex permeability, respectively.

S1.2 Impedance Matching and Attenuation Constant

The impedance matching degree between absorbers and free space determines whether the electromagnetic wave (EMW) can be propagated to the interior of the absorber. Specifically, the impedance of microwave absorbers should be infinitely close to that of free space. The impedance matching can be revealed by delta functions as follows [S3]:

$$|\Delta| = |\sin h^2 (Kfd) - M| \quad \text{(S3)}$$

in which, the K and M are calculated according to the complex permittivity and permeability as expressed as:

$$K=\frac{4\pi\sqrt{\mu'\varepsilon'}\sin\frac{\delta_e+\delta_m}{2}}{c\cos\delta_e\cos\delta_m} \tag{S4}$$

$$M=\frac{4\mu'\cos\delta_e\varepsilon'\cos\delta_m}{(\mu'\cos\delta_e-\varepsilon'\cos\delta_m)^2+\left[\tan\left(\frac{\delta_m}{2}-\frac{\delta_e}{2}\right)\right]^2(\mu'\cos\delta_e+\varepsilon'\cos\delta_m)^2} \tag{S5}$$

The smaller value of $|\Delta|$ represents more excellent impedance matching without excessive reflection of EMW.

In addition, the attenuation constant α is another vital factor for MA, determining the attenuation ability of the absorbers to incident EMW, which can be described as following equations [S4]:

$$\alpha=\frac{\pi f}{c}\sqrt{\mu'\varepsilon'}\sqrt{2\left[\tan\delta_e\tan\delta_m-1+\sqrt{(1+\tan^2\delta_e+\tan^2\delta_m+\tan^2\delta_e\delta_m)}\right]}$$

$$=\frac{\sqrt{2}\pi f}{c}\left[(\mu''\varepsilon''-\mu'\varepsilon')+\sqrt{(\mu''\varepsilon''-\mu'\varepsilon')^2+(\mu'\varepsilon''+\mu''\varepsilon')^2}\right]^{1/2} \tag{S6}$$

S1.3 Debye Relaxation

According to the Debye theory, the relative complex permittivity can be expressed as follows [S2, S5]:

$$\varepsilon_r=\varepsilon'-j\varepsilon''=\varepsilon_\infty+\frac{\varepsilon_s-\varepsilon_\infty}{1+j\omega\tau} \tag{S7}$$

Where ε_s is the static dielectric constant, ε_∞ is the dielectric constant at infinite frequency, $\omega=2\pi f$ is the angular frequency, and τ refer to the polarization relaxation time. In consequence, the ε' and ε'' can be described as follows:

$$\varepsilon'=\varepsilon_\infty+\frac{\varepsilon_s-\varepsilon_\infty}{1+\omega^2\tau^2} \tag{S8}$$

$$\varepsilon''=\frac{\varepsilon_s-\varepsilon_\infty}{1+\omega^2\tau^2}\omega\tau+\frac{\sigma}{\omega\varepsilon_0}=\varepsilon_p''+\varepsilon_c'' \tag{S9}$$

Based on the **Eqs. S8** and **S9**, the relationship between ε' and ε'' can be expressed as follows:

$$\left(\varepsilon'-\frac{\varepsilon_s+\varepsilon_\infty}{2}\right)^2+(\varepsilon'')^2=\left(\frac{\varepsilon_s-\varepsilon_\infty}{2}\right)^2 \tag{S10}$$

S1.4 Calculation of Specific Reflection Loss

Specific reflection loss (SRL) is proposed to compare the effectiveness of microwave absorbers. Taking into account the matching thickness, density, and absorption intensity. Mathematically, SRL can be obtained by dividing the RL value with filler loading and matching thickness of absorbers as follows [S6]:

$$SRL=\frac{RL}{filler\ loading\times matching\ thickness} \tag{S11}$$

S2 Supplementary Figures and Tables

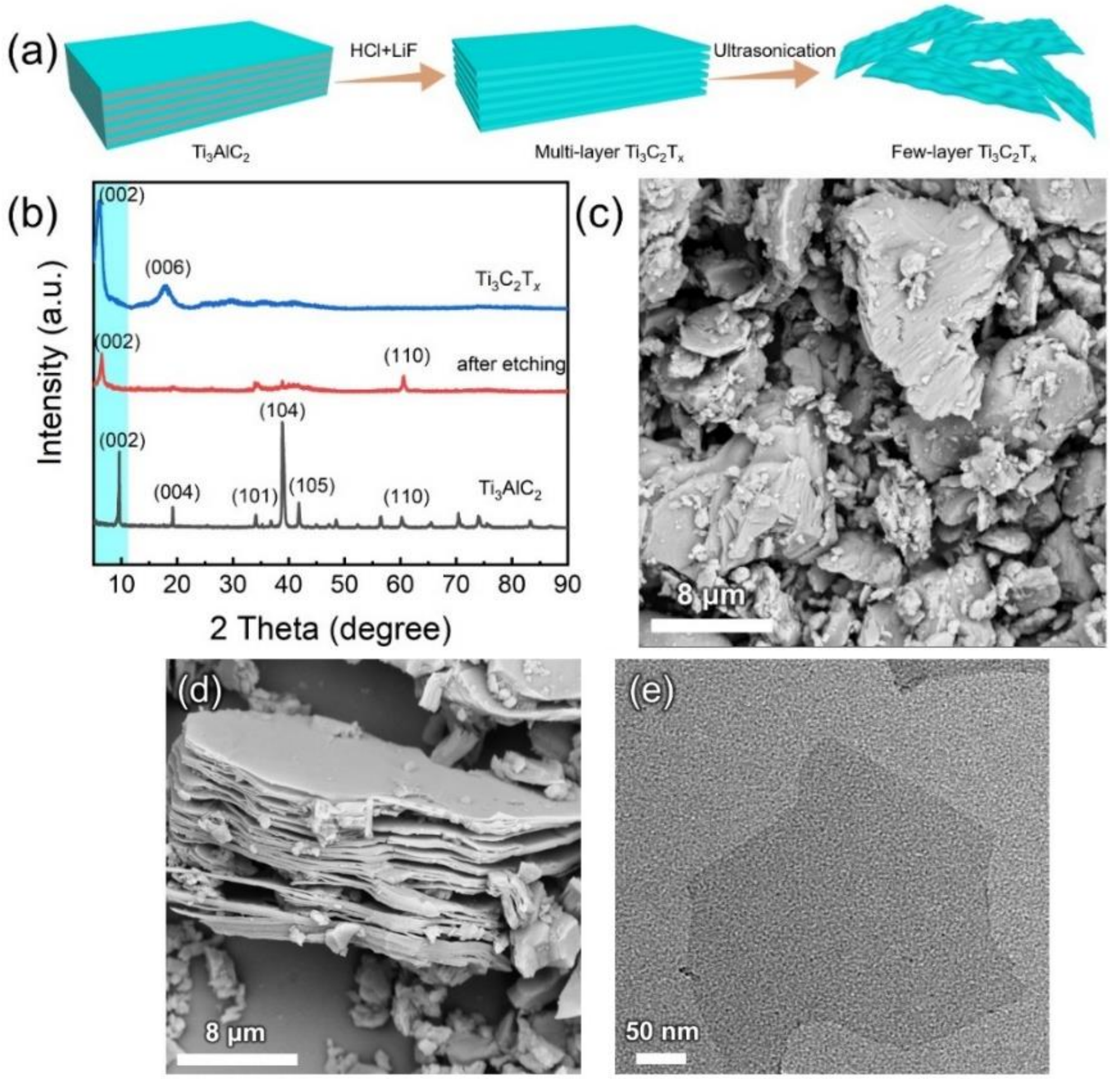

Fig. S1 Structural evolution from Ti_3AlC_2 MAX to $Ti_3C_2T_x$ MXene. (**a**) Schematic illustration of the synthesis process of $Ti_3C_2T_x$ MXene nanosheets; (**b**) XRD patterns of raw Ti_3AlC_2, multi-layer $Ti_3C_2T_x$ MXene etched by HCl/LiF and few-layer $Ti_3C_2T_x$ MXene after ultrasonication process; SEM images for raw Ti_3AlC_2 (**c**) and multi-layer $Ti_3C_2T_x$ MXene (**d**); TEM image of the few-layer $Ti_3C_2T_x$ nanosheets(**e**)

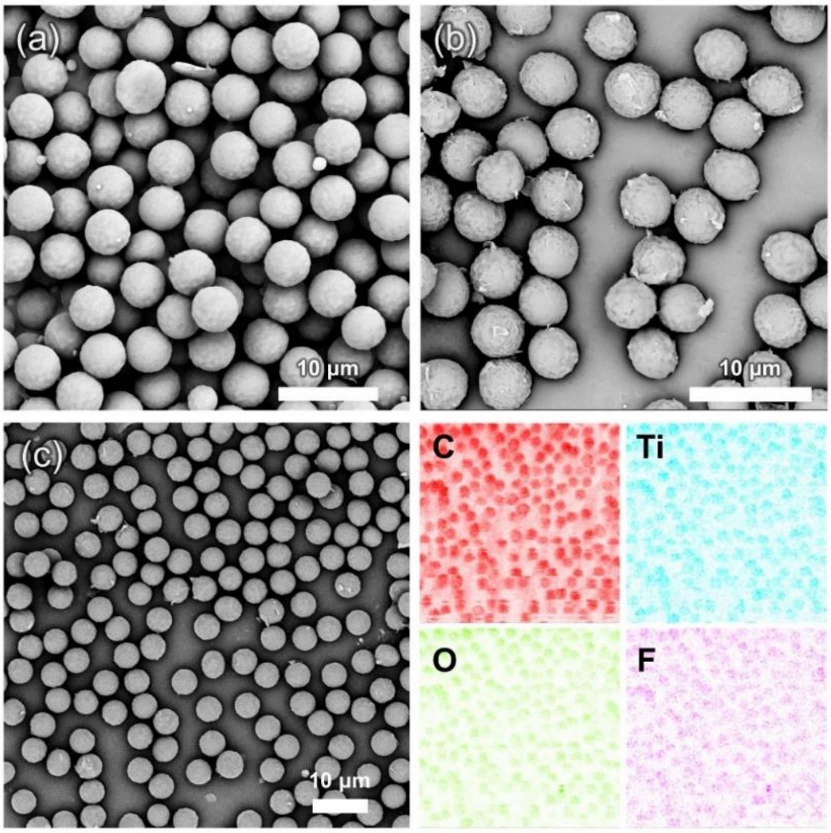

Fig. S2 SEM images for PMMA microspheres (**a**) and PMMA@$Ti_3C_2T_x$ composite microspheres (**b**), as well as corresponding elemental Mapping of PMMA@$Ti_3C_2T_x$ microspheres (**c**)

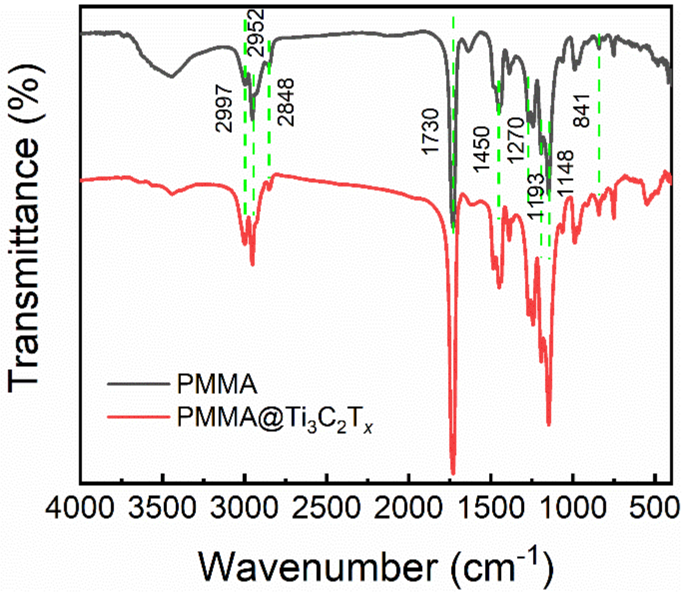

Fig. S3 FTIR spectra of PMMA and PMMA@$Ti_3C_2T_x$

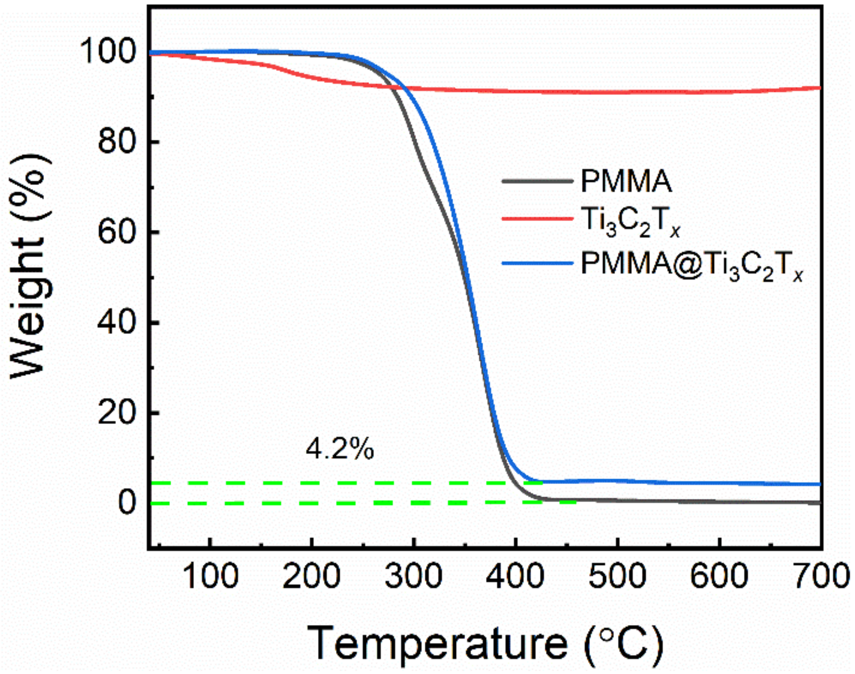

Fig. S4 TGA curves of PMMA, $Ti_3C_2T_x$ and PMMA@$Ti_3C_2T_x$ in N_2 atmosphere

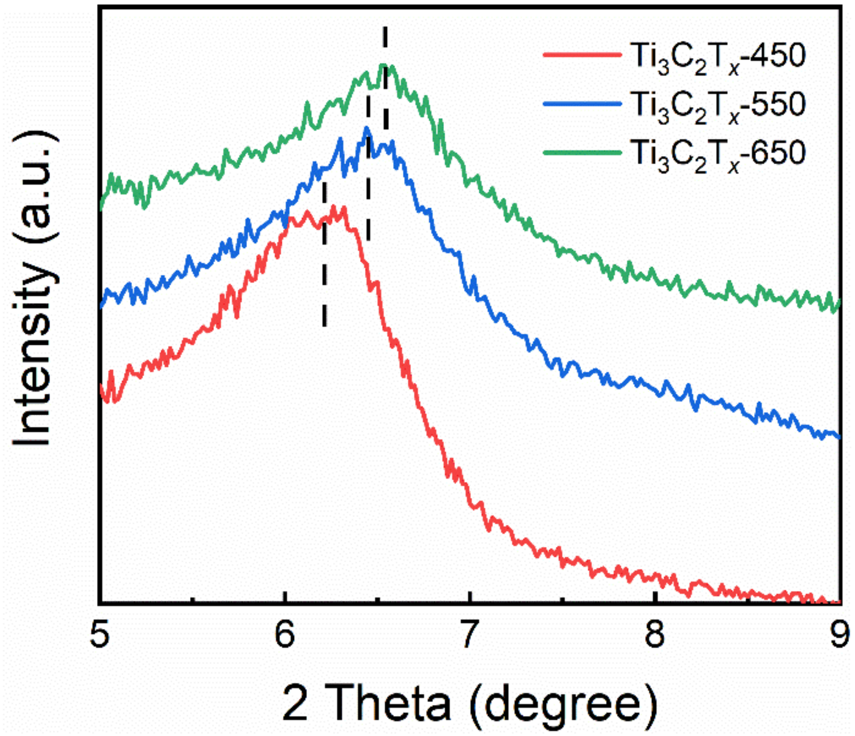

Fig. S5 XRD patterns of $Ti_3C_2T_x$ hollow spheres

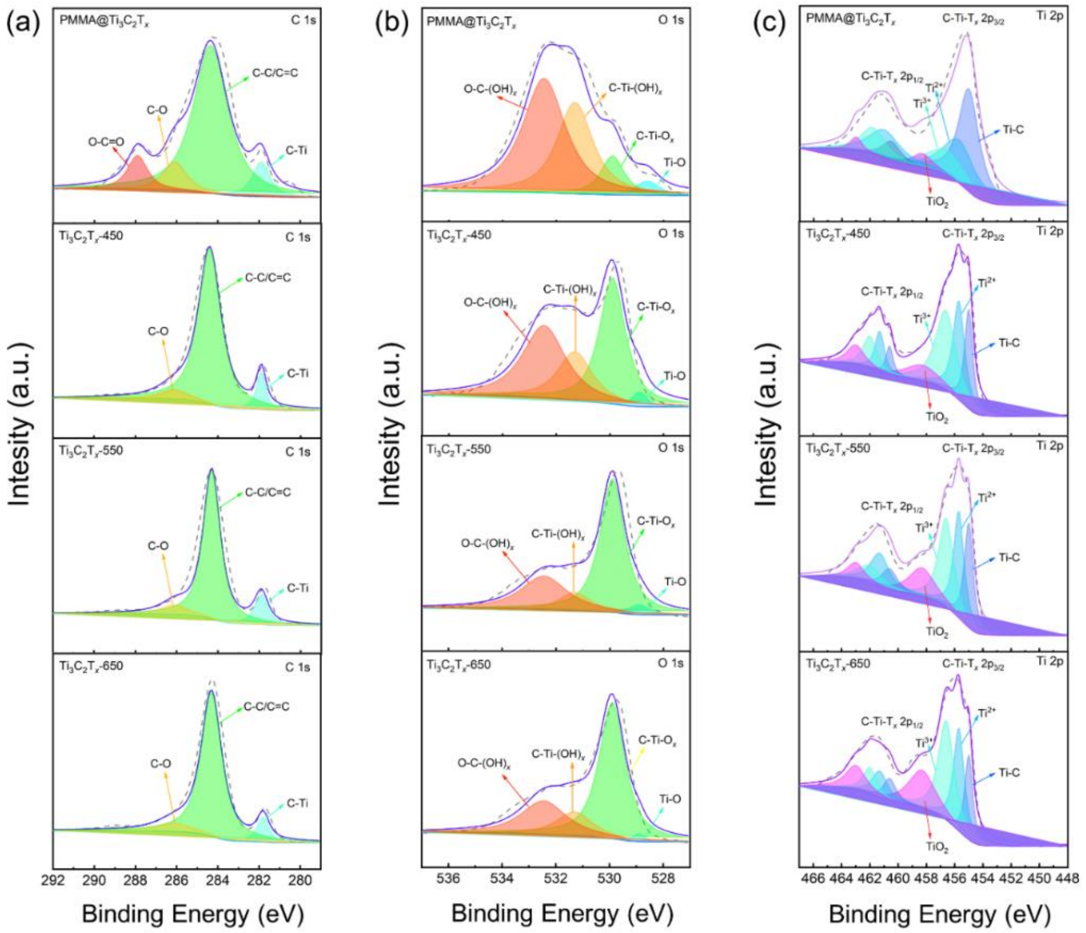

Fig. S6 High-resolution XPS spectra of PMMA@$Ti_3C_2T_x$, $Ti_3C_2T_x$-450, $Ti_3C_2T_x$-550, and $Ti_3C_2T_x$-650, (**a**) C 1s, (**b**) O 1s, (**c**) Ti 2p

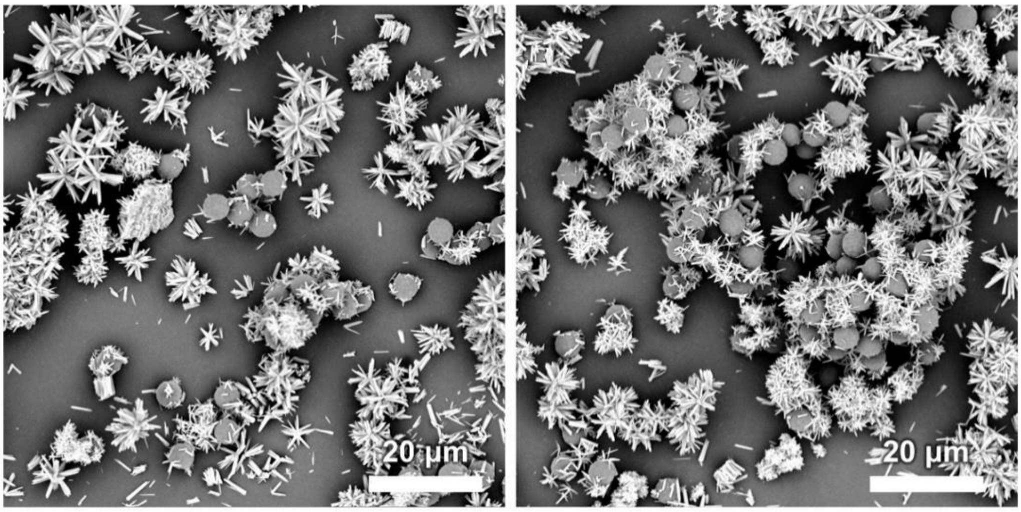

Fig. S7 SEM images of PMMA/ZnO

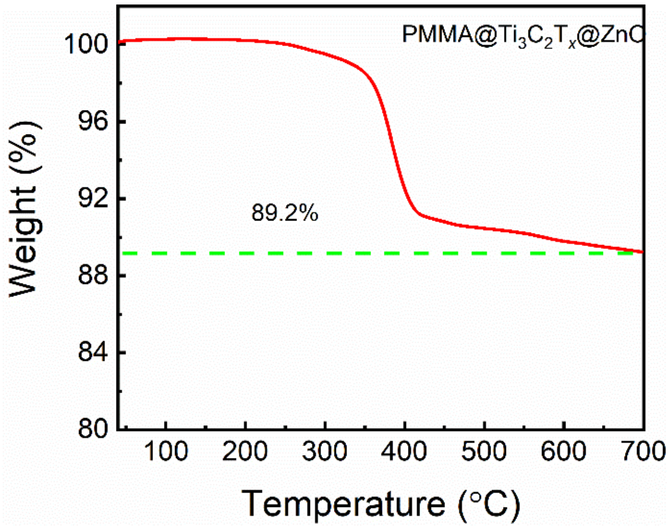

Fig. S8 TGA curves of PMMA@$Ti_3C_2T_x$@ZnO in N_2 atmosphere

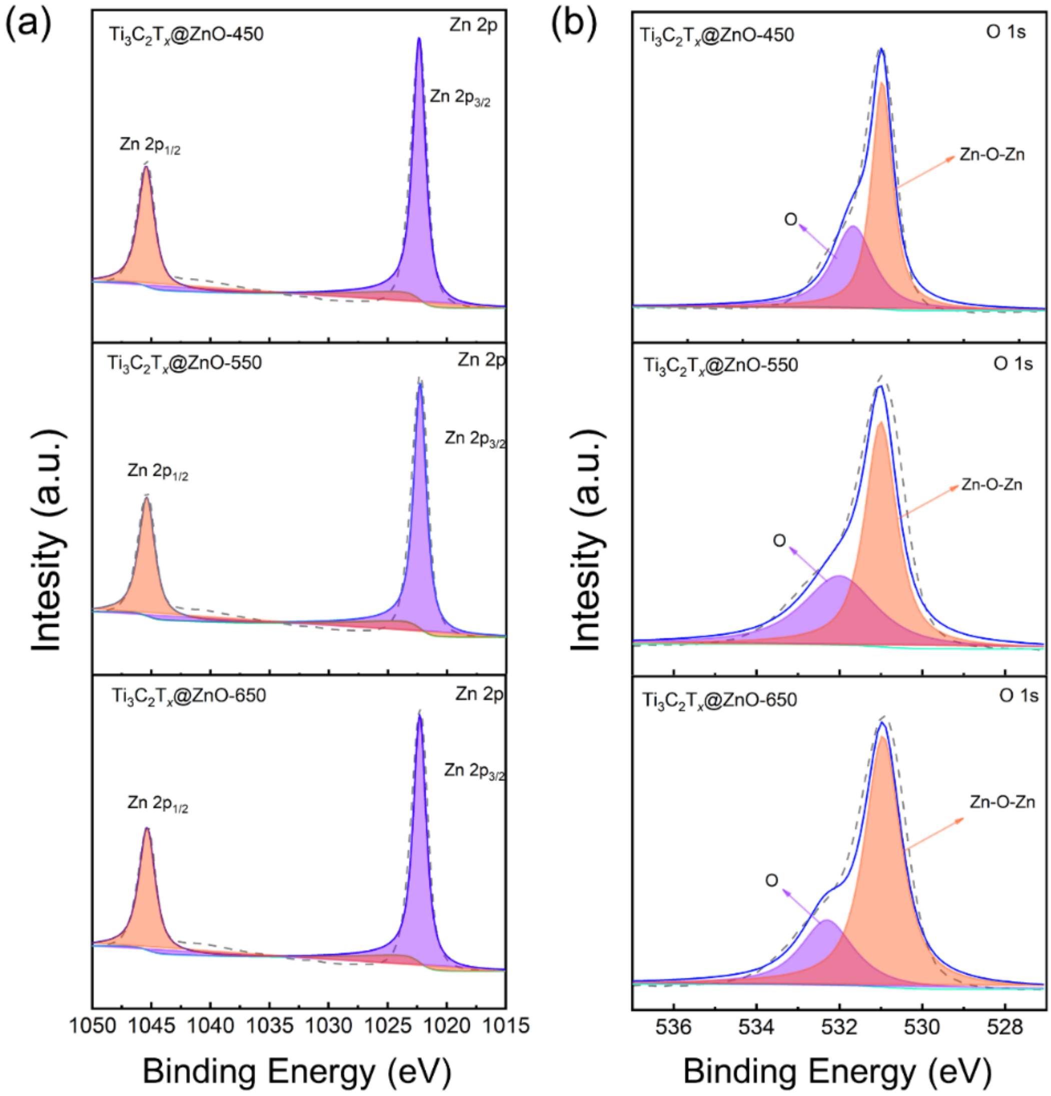

Fig. S9 High-resolution XPS spectra of $Ti_3C_2T_x$@ZnO-450, $Ti_3C_2T_x$@ZnO-550, and $Ti_3C_2T_x$@ZnO-650, (**a**) Zn 2p, (**b**) O 1s

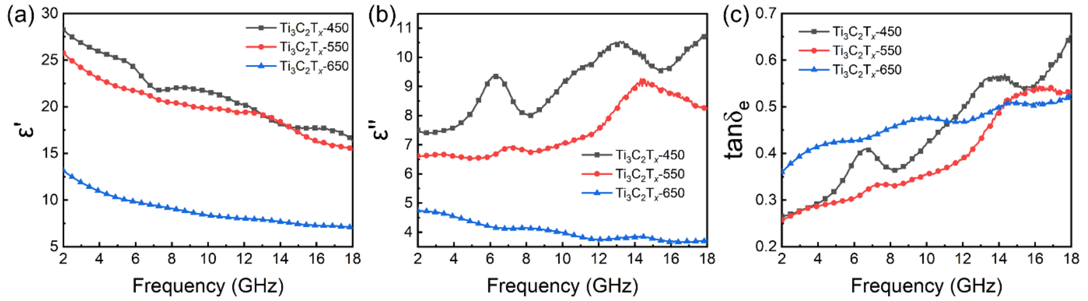

Fig. S10 Frequency dependence of (**a**) real part ε′, (**b**) imaginary part ε″, and (**c**) dielectric loss tangents of complex permittivity of $Ti_3C_2T_x$ hollow spheres

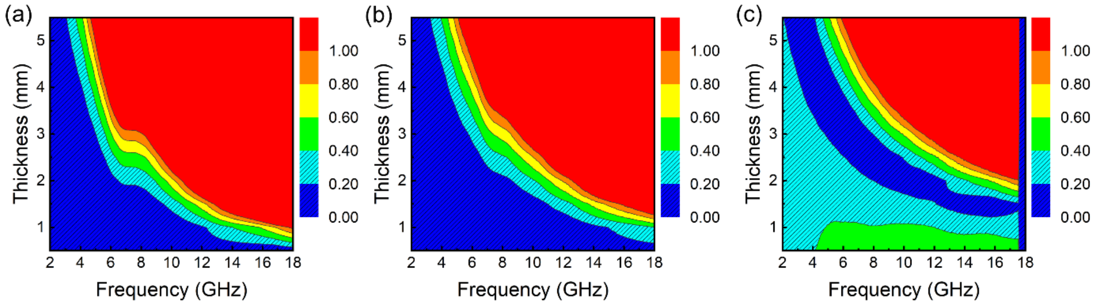

Fig. S11 Calculated delta value maps of (**a**) $Ti_3C_2T_x$-450, (**b**) $Ti_3C_2T_x$-550, and (**c**) $Ti_3C_2T_x$-650

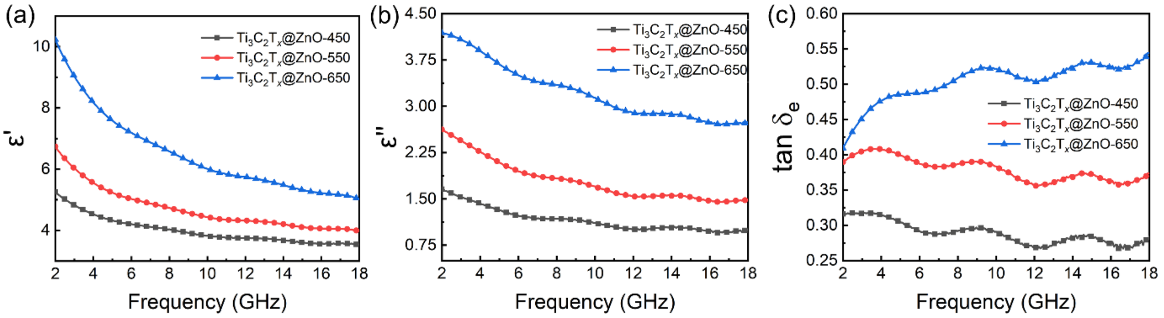

Fig. S12 Frequency dependence of (**a**) real part ε′, (**b**) imaginary part ε″, and (**c**) dielectric loss tangents of complex permittivity of $Ti_3C_2T_x$@ZnO hollow spheres

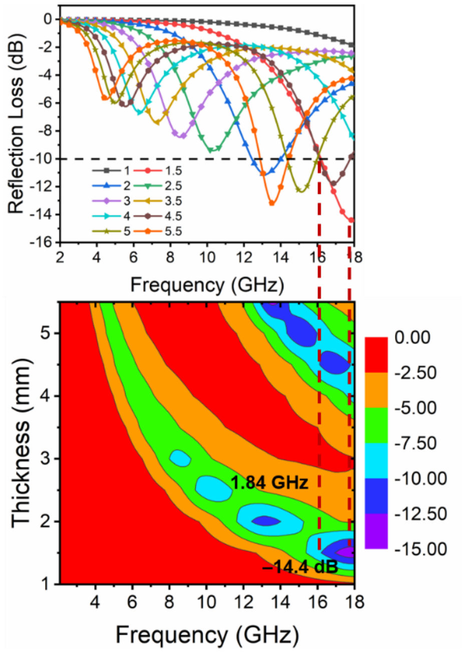

Fig. S13 Frequency dependence of reflection loss for ZnO with the filler loading of 40 wt%

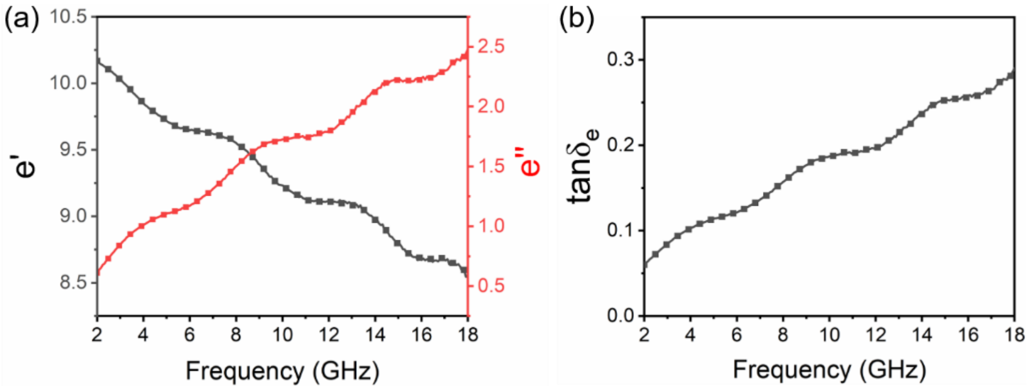

Fig. S14 Frequency dependence of (**a**) real part ε′ and imaginary part ε″, and (**b**) dielectric loss tangents of complex permittivity of ZnO

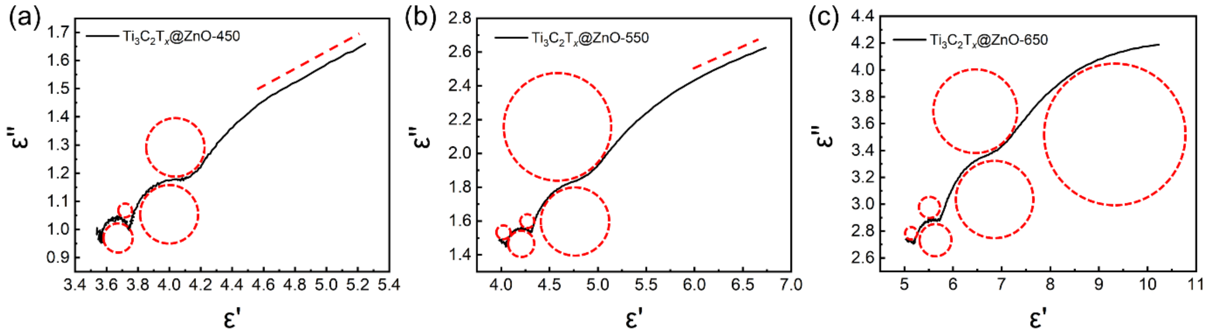

Fig. S15 Cole−Cole semicircles of (**a**) $Ti_3C_2T_x$@ZnO-450, (**b**) $Ti_3C_2T_x$@ZnO-550, and (**c**) $Ti_3C_2T_x$@ZnO-650

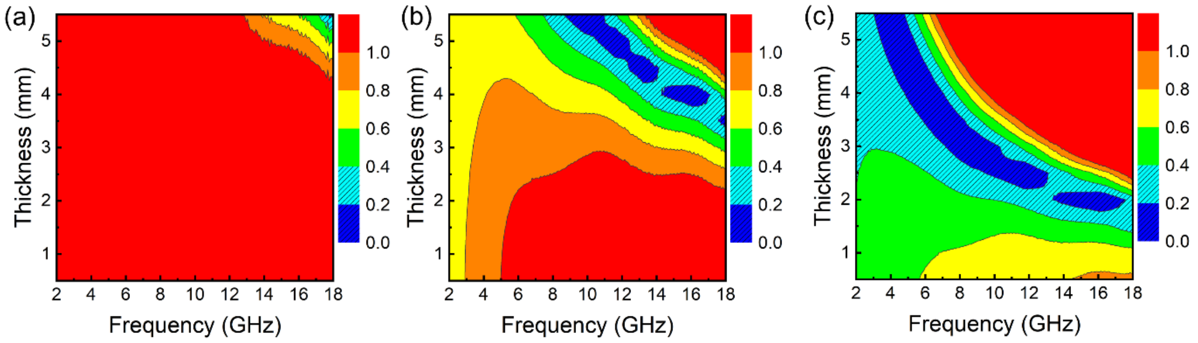

Fig. S16 Calculated delta value maps of (**a**) $Ti_3C_2T_x$@ZnO-450, (**b**) $Ti_3C_2T_x$@ZnO-550, and (**c**) $Ti_3C_2T_x$@ZnO-650

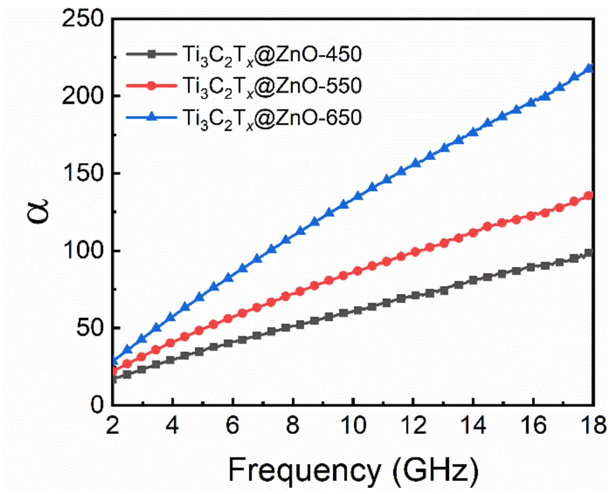

Fig. S17 Attenuation constants of $Ti_3C_2T_x$@ZnO hollow spheres

Table S1 Comparison for the EMW adsorption properties of ZnO-based EMW absorbers reported in previous literatures (assume that the mass of each sample is 100 mg)

**Samples**	**RL_{min} (dB)**	**Filler loading (wt%)**	**Thickness (mm)**	**EAB (GHz)**	**SRL (dB mm^{-1} mg^{-1})**	**Refs**
GS-ZnO	–45.05	50	2.2	2.5	–40.95	[S7]
ZnO-MXene	–26.3	75	4	1.4	–8.76	[S8]
CF-$Ti_3C_2T_x$/Ni/ZnO	–35.1	100	2.8	~3	–12.53	[S9]
ZnO@C	–50.05	60	2	5.76	–41.70	[S10]
MnO_2/ZnO	–41.3	40	5.4	~1.5	–19.12	[S11]
ZnO/Fe_3O_4	–36.23	60	2.7	4.02	–22.36	[S12]
Ni@ZnO	–30.2	50	2.2	2.5	–27.45	[S13]
FeCo/ZnO	–34.8	60	1.5	5.1	–38.66	[S14]
CH/ZnO	–54.68	50	3.21	1.0	–34.06	[S15]
C/$NiCo_2O_4$/ZnO	–43.61	60	2.4	4.32	–30.28	[S16]
$Ti_3C_2T_x$@ZnO-650	–57.4	40	2	4.24	–71.75	This work

Supplementary References

[S1] L. Liang, Q. Li, X. Yan, Y. Feng, Y. Wang et al., Multifunctional magnetic $Ti_3C_2T_x$ MXene/graphene aerogel with superior electromagnetic wave absorption performance. ACS Nano **15**(4), 6622-6632 (2021). https://doi.org/10.1021/acsnano.0c09982

[S2] Y. Wang, H. Wang, J. Ye, L. Shi, X. Feng, Magnetic CoFe alloy@C nanocomposites derived from ZnCo-MOF for electromagnetic wave absorption. Chem. Eng. J. **383**, 123096 (2020). https://doi.org/10.1016/j.cej.2019.123096

[S3] N. Yang, Z.X. Luo, S.C. Chen, G. Wu, Y.Z. Wang, Fe_3O_4 nanoparticle/N-doped carbon hierarchically hollow microspheres for broadband and high-performance microwave absorption at an ultralow filler loading. ACS Appl. Mater. Interfaces **12**(16), 18952-18963 (2020). https://doi.org/10.1021/acsami.0c04185

[S4] J.B. Cheng, Y.Q. Wang, A.N. Zhang, H.B. Zhao, Y.Z. Wang, Growing MoO_3-doped WO_3 nanoflakes on rGO aerogel sheets towards superior microwave absorption. Carbon **183**, 205-215 (2021). https://doi.org/10.1016/j.carbon.2021.07.019

[S5] H. Wang, F. Meng, F. Huang, C. Jing, Y. Li et al., Interface modulating CNTs@PANi hybrids by controlled unzipping of the walls of CNTs to achieve tunable high-performance microwave absorption. ACS Appl. Mater. Interfaces **11**(12), 12142-12153 (2019). https://doi.org/10.1021/acsami.9b01122

[S6] Y. Li, X. Liu, X. Nie, W. Yang, Y. Wang et al., Multifunctional organic–inorganic hybrid aerogel for self-cleaning, heat-insulating, and highly efficient microwave absorbing material. Adv. Funct. Mater. **29**(10), 1807624 (2019).

https://doi.org/10.1002/adfm.201807624

[S7] M. Han, X. Yin, L. Kong, M. Li, W. Duan et al., Graphene-wrapped ZnO hollow spheres with enhanced electromagnetic wave absorption properties. J. Mater. Chem. A **2**(39), 16403-16409 (2014). https://doi.org/10.1039/c4ta03033h

[S8] Y. Qian, H. Wei, J. Dong, Y. Du, X. Fang et al., Fabrication of urchin-like ZnO-MXene

nanocomposites for high-performance electromagnetic absorption. Ceram. Int. **43**(14), 10757-10762 (2017). https://doi.org/10.1016/j.ceramint.2017.05.082

[S9] S. Wang, D. Li, Y. Zhou, L. Jiang, Hierarchical $Ti_3C_2T_x$ MXene/Ni chain/ZnO array hybrid nanostructures on cotton fabric for durable self-cleaning and enhanced microwave absorption. ACS Nano **14**(7), 8634-8645 (2020). https://doi.org/10.1021/acsnano.0c03013

[S10] L. Yan, M. Zhang, S. Zhao, T. Sun, B. Zhang et al., Wire-in-tube ZnO@carbon by molecular layer deposition: accurately tunable electromagnetic parameters and remarkable microwave absorption. Chem. Eng. J. **382**, 122860 (2020). https://doi.org/10.1016/j.cej.2019.122860

[S11] G. He, Y. Duan, H. Pang, J. Hu, Superior microwave absorption based on ZnO capped MnO_2 nanostructures. Adv. Mater. Interfaces **7**(15), 2000407 (2020). https://doi.org/10.1002/admi.202000407

[S12] W. Ma, R. Yang, T. Wang, ZnO nanorod-based microflowers decorated with Fe_3O_4 nanoparticles for electromagnetic wave absorption. ACS Appl. Nano Mater. **3**(8), 8319-8327 (2020). https://doi.org/10.1021/acsanm.0c01728

[S13] J. Deng, Q. Wang, Y. Zhou, B. Zhao, R. Zhang, Facile design of a ZnO nanorod–Ni core–shell composite with dual peaks to tune its microwave absorption properties. RSC Adv. **7**(15), 9294-9302 (2017). https://doi.org/10.1039/c6ra28835a

[S14] X. Bao, X. Wang, X. Zhou, G. Shi, G. Xu et al., Excellent microwave absorption of FeCo/ZnO composites with defects in ZnO for regulating the impedance matching. J. Alloys Compd. **769**, 512-520 (2018). https://doi.org/10.1016/j.jallcom.2018.08.036

[S15] Y. Qi, L. Qi, L. Liu, B. Dai, D. Wei et al., Facile synthesis of lightweight carbonized hydrochars decorated with dispersed ZnO nanocrystals and enhanced microwave absorption properties. Carbon **150**, 259-267 (2019). https://doi.org/10.1016/j.carbon.2019.05.026

[S16] J. Fan, W. Xing, Y. Huang, J. Dai, Q. Liu et al., Facile fabrication hierarchical urchin-like $C/NiCo_2O_4/ZnO$ composites as excellent microwave absorbers. J. Alloys Compd. **821**, 153491 (2020). https://doi.org/10.1016/j.jallcom.2019.153491
